# A diverse Palaeoproterozoic microbial ecosystem implies early eukaryogenesis

**DOI:** 10.1098/rstb.2024.0092

**Published:** 2025-08-07

**Authors:** Emmanuelle J. Javaux

**Affiliations:** ^1^ Early Life Traces and Evolution-Astrobiology R.U., Université de Liège, Liege, Belgium

**Keywords:** cellular palaeobiology, eukaryogenesis, evolution, microbial symbiosis, protist, cyanobacteria, organic-walled microfossils, Palaeoproterozoic, predation

## Abstract

Microbial interactions may lead to major events in life and planetary evolution, such as eukaryogenesis, the birth of complex nucleated cells. In synergy with microbiology, cellular palaeobiology may shed some light on this very ancient and debated affair and its circumstances. The 1.78–1.73 Ga McDermott Formation, McArthur Basin (Australia), preserves a microfossil assemblage that provides unique insights into the evolution of early eukaryotes. The fossil cells display a level of morphological complexity, disparity and plasticity requiring a complex cytoskeleton and an endomembrane system, pushing back the minimum age of uncontested eukaryotic fossils by more than 100 million years (Ma). They also document an earlier appearance of reproduction by budding, simple multicellularity and diverse programmed openings of cyst wall implying a life cycle, as well as possible evidence for microbial symbiosis and behaviour, including eukaryovory and ectosymbiosis. This microbial community that also includes cyanobacterial cells preserving thylakoids, microbial mats and other microfossils, thrived in supratidal to intertidal marine environments with heterogeneous but mostly suboxic to anoxic redox conditions. Taken together, these observations imply early eukaryogenesis, including mitochondrial endosymbiosis in micro-/nano-oxic niches, and suggest a >1.75 Ga minimum age for the Last Eukaryotic Common Ancestor (LECA), preceded by a deeper history of the domain Eukarya, consistent with several molecular clocks and the fossil record.

This article is part of the discussion meeting issue ‘Chance and purpose in the evolution of biospheres’.

## 1. Introduction

Since its origin, microbial life has been the dominant form of life and it controls Earth's biogeochemical cycles [1]. Communities of microbial cells closely interact continuously and these symbioses, which can be beneficial, detrimental or neutral for the partners involved, are ubiquitous and ecologically important [2]. They appear as one of the fundamental mechanisms leading to critical evolutionary transitions, such as eukaryogenesis and plastid endosymbiosis. Complementary to microbiology, cellular palaeobiology—the study of fossil cells—may provide direct evidence of these very ancient events that have biospheric and planetary scale consequences, as well as inform on their minimum age and ecological context.

### (a) Eukaryogenesis

The birth of complex nucleated cells, a process called eukaryogenesis, arose from the association of prokaryotic archaeal and bacterial cells, including an Asgard archaea, an alphaproteobacterium ancestor of the mitochondrion, and perhaps other bacterial partners [3–21]. Members of PVC (Planctomycetes, Verrucomicrobia and Chlamydiae) bacteria can form protrusions and have membrane coat proteins sustaining their prokaryotic endomembrane system and other eukaryotic-like features that led to suggest also their implication in eukaryogenesis [3,22]. This symbiosis, whatever the proposed model of eukaryogenesis, led to one of the most radical changes in life evolution, a third domain of life with a new scale of cellular complexity and excitability [23,24]. However, the sequence of evolutionary events occurring between FECA (the First Eukaryotic Common Ancestor) and the Last Eukaryotic Common Ancestor (LECA), and the prokaryotic partners involved, as well as the environment where it took place are debated. To date, the timing and relative order of evolution of cellular features characterizing LECA, such as the nucleus, endomembranes, cytoskeleton and mitochondrion, are unknown [12–15]. Eukaryogenesis scenarios also vary according to the processes (phagocytosis, endocytosis by invaginations or capture by protrusions) and timing of (early, mid, late) acquisition of the mitochondrion.

Analyses of the Asgaard Archaeal genomes suggest that some cellular complexity such as the cytoskeleton and vesicle trafficking may have emerged before other eukaryotic traits of LECA [25,26]. Two species of Archaea of the suborder Lokiarchaeales in culture that harbour eukaryotic-like genes and a dynamic actin cytoskeleton which extends throughout the membrane protrusions from the few 100 nm’s cell bodies [27,28] show that these prokaryotes may show complex morphologies that could have facilitated eukaryogenesis [27]. Another (uncultivated) archaeon from the order Hodarchaeales, which was recently proposed as the sister lineage of eukaryotes with a unique set of eukaryotic signature proteins [10], has an elongated cell body prolonged by a rounded expansion at one pole with a localized DNA [29]. These cells are 1.5–5.2 μm long, and 1.1−0.8 μm in width for the round expansion and the elongate cell body respectively. The authors suggested that their relatively large size and complex morphology could resemble a transitional stage in eukaryogenesis [29]. However, it is unknown if these modern complex archaea present ancestral or derived features after more than 2 billion years of evolution from the archaeal ancestor of eukaryotes [10]. A recent study of two new strains of Hodarchaeales shows that they do not contain endomembranes and that only non-dividing cells produce complex actin-containing protrusions, modifying their cell size. The number and volume of protrusions increase with increasing availability of carbon and energy, suggesting that they are used both for syntrophy and for nutrient, energy and building block storage for later cell division [30]. These authors propose that FECA was a simple-celled anaerobic syntrophic peptide-degrading archaeon, possibly with genes for oxygen tolerance (detoxification) but not for aerobic respiration, and with a behaviour focused on cell maintenance and construction rather than division. This differs from most prokaryotes, which devote an increasing fraction of their entire metabolism to growth with increasing cell size, whereas eukaryotes devote a diminishing fraction [31]. Therefore, the transition from archaea to eukaryote may have been sharper than previously assumed, both in cell ultrastructure and aerobiosis [30].

Several recent studies [32,33] highlight the large contribution of both archaea and alphaproteobacteria genomes in eukaryotic metabolisms, in contradiction to the view that eukaryotic metabolism is predominantly of bacterial origin and in support of syntrophic scenarios on the origin of the eukaryotic cell. Waves of lateral gene transfers (LGT) from other bacteria and viruses are proposed, with some likely preceding the mitochondrial endosymbiosis [34]. A mosaic origin of eukaryotic metabolism was also proposed with the Chlamydial contribution to anaerobic metabolism [35]. The adaptation to hypoxia by protists originated from genes transferred from bacteria to various eukaryote lineages by multiple independent events of LGT, well after the establishment of mitochondria within eukaryotes [36]. Sterol synthesis originated from myxobacteria [37].

Although the environment where eukaryogenesis took place is not known, it must have happened close to an oxygen source [38]. Photosynthesizing microbial mats seem a suitable habitat, where cyanobacteria produced oxygen in surface layers and where close microbial interactions facilitate gene, nutrient and electron transfers through light- and redox-stratified layers [39]. In such habitat, the archaeon–mitochondrial symbiont association could use some oxygen to support aerobic respiration, to maintain a fully functional oxygen-dependent electron transport chain originated from the alphaproteobacterial endosymbiont over generations [40,41] and to produce sterols [37]. Alternatively, Mills *et al.* [42] propose that eukaryogenesis occurred in anoxic environments, such as methane-rich marine sediments or an anoxic water column, and that later eukaryotic lineages migrated to shallower oxygenated settings in the mid-Proterozoic.

Archaea are not capable of phagocytosis despite having elements of a complex cytoskeleton and vesicle trafficking [43]. However, other processes besides phagocytosis, an early evolving process known in many protists including the recently defined Provora [44], may permit the acquisition of endosymbionts and are invoked in several eukaryogenesis models (e.g. [12,27]) such as protrusion formation (entangling) illustrated by cultivated Asgard archaea [27,28,30] and blebs (membrane bulging) known in several archaea [5] and membrane invagination in one bacterium [45]. This ‘phagocytosing’ plantomycete bacterium is able to digest bacteria and protists preys by invagination of its outer and cytoplasmic membranes, resulting in digestion in specific compartments. Despite the presence of an archaeon-derived actin-like gene, the process is not homologous to eukaryotic phagocytosis and is unique (so far) in prokaryotes [45]. These examples and others such as the multiple parallel origins of endosymbionts in *Euplotes* ciliates suggest that symbiotic events leading to organelles may be more common in evolution than previously thought and can happen rapidly [2]. Such symbioses may provide different initial metabolic, nutritional, defence, mobility or other benefits depending on the host and symbiont origins and on the conditions when and where they occur. Similarly, during eukaryogenesis, several stem lineages with permanent cellular innovations could have emerged before LECA [16,38,46].

Sometime after eukarogenesis and the birth of LECA, another organelle, the plastid, emerged from an endosymbiotic event of a *Gloeomargarita*-like cyanobacterium in a freshwater environment [7,47,48] where it was acquired by an early heterotrophic protist (reviews in [ 49,50]). This event led to the diversification of green and red algae more than 1 Ga [51–53] to possibly 1.6 Ga ago [54,55], and later to land plants, causing a profound change in Earth systems. This uptake could have involved phagocytosis without subsequent digestion, or invagination of the plasma membrane perhaps preceded by prolonged ectosymbiosis, and provided a source of molecular oxygen and carbohydrates to the protist host [49]. This primary endosymbiosis happened a second time independently and very recently (90–140 Ma, [56]) by phagocytosis of *Prochlorococcus* and *Synechococcus* cyanobacterial clades that became the photosynthetic organelles (‘chromatophores’) of the euglyphid amoeba *Paulinella*, perhaps facilitating low-light photosynthesis [49]. Subsequent secondary and tertiary endosymbiotic events, where microalgae were acquired by heterotrophic protists, led to the spread of oxygenic photosynthesis in various clades of eukaryotes, again modifying trophic chains in oceans and on land.

### (b) The last eukaryotic common ancestor (LECA)

A consensus view [17] infers the following features for the population of cells that constituted LECA and resembled modern heterotrophic flagellate protists: a nucleus, nucleolus, chromatin and nuclear pore complexes; DNA of multiple origins (eukaryotic, archaeal, bacterial, viral) and linear nuclear chromosomes with centromeres and telomeres and a complex set of genetic regulation systems; a complex actin- and tubulin microtubule-based cytoskeleton with associated motor proteins; flagella, pseudo/filopodia; actin-based endo- and exocytosis; mitosis, meiosis and a facultative sexual cycle; a complex and diversified endomembrane and endomembrane trafficking system (an endoplasmic reticulum and Golgi apparatus); membranes composed of fatty acid chains linked to a glycerol-3-phosphate (G3P) head group via ester bonds and containing diverse sterols; peroxisomes (involved in regulation of mitochondrial-generated reactive oxygen species (ROS) and oxidation of fatty acids [57]); vacuoles; and a fully integrated mitochondrial organelle.

Mapping phenotypic traits onto a new phylogeny of heterotrophic flagellates, which are the most diverse eukaryotes and present in almost all of the major eukaryotic supergroups, and the main grazers of bacterio- and phytoplankton in the ocean [58,59], led to inferring a biflagellate ancestor with an excavate-like feeding groove [60]. Williamson *et al*. [61] propose a new tree of eukaryotes based on mitochondrial genomes and confirm their excavate ancestry, placing the root (LECA) between two multi-supergroup assemblages, the Opimoda (Amoebozoa, Obazoa, CRuMS and other protist supergroups) and the Biphoda (Archaeplastida, Haptophyta, SAR, Provora and other protist supergroups). Because excavate taxa are placed on both sides of the root, their traits are ancestral to all eukaryotes and to LECA. LECA is thus inferred to have been a small (25 µm or less) unicellular aerobe phagotrophic predatory flagellate, with a typical flagellate excavate morphology [60–63], feeding on prey in suspension, possessing mitochondria capable of oxidative phosphorylation and a complex cytoskeleton of microtubular and non-microtubular elements. In modern excavates that are slow swimmers, the cell is attached with an anterior flagellum to a surface and feeding through ‘foraging phagocytosis’ using a posterior flagellum equipped with vanes (extensions). The vaned flagellum generates a current and beats into a ventral groove and concentrates bacteria and picoplankton. Phagocytosis is facilitated by a moving ‘wave’ of the cell membrane that sweeps posteriorly along the groove [63]. Such excavate-like cellular organization (with a central groove for feeding and flagella) appears more common than previously thought with the recent discovery of diverse small (3–10 µm) predatory flagellates in aquatic environments that can feed by phagocytosing a whole prey or by nibbling on prey, leading to the creation of the new supergroup Provora [44]. Provora are diverse globally distributed deep-branching eukaryotes [44,64,65]. Small bacterial or eukaryotic prey cells (<10 µm) are consumed by whole-cell phagocytosis, while larger prey (>10 µ) are eaten by ‘nibbling’ and by vampire-like cytoplasmic sucking called ‘myzocytosis’ [65].

### (c) Oxygen and early eukaryotes

LECA is considered as an aerobe mitochondriate protist (except for one study [62], but see [17,40]). A recent paper suggests early aerobiosis in archaea [16] although it might be instead interpreted as oxygen tolerance [30] and it is uncontested that LECA acquired aerobiosis from the alphaproteobacterial ancestor of the mitochondrion [38,48] and that anaerobiosis was acquired by LGT from Chlamydial bacteria [35]. Also, both LECA and pre-LECA eukaryotes acquired steroid biosynthesis genes from aerobe myxobacteria that used molecular oxygen [37]. Thus, molecular oxygen was required in eukaryogenesis that could have taken place in a redox-stratified photosynthetic microbial mat where oxygenic photosynthesis released oxygen ([39,41]; but see [42] for an alternative view).

The geological record and molecular phylogenies indicate that some abiotic and/or biological molecular oxygen was available to life long before the permanent oxygenation of the Earth between 2.4 and 2.1 Ga (the Great Oxidation Event [66,67]) preceded by possible but debated earlier ‘oxygen whiffs’ [42,68–71] or oases [72] (but see [73,74]). The evolution of most redox-sensitive and O_2_-utilizing protein families is dated in the Archean, before the GOE, possibly around approximately 3.33−2.85 Ga [75,76], as previously suggested [77]. Based on respiratory and oxygen tolerance genes and bacterial habitat, the earliest aerobic bacteria emerged in the Archaean, predating the GOE by 900 Ma [78]. Moreover, photosystem II, the water-oxidizing and O_2_-evolving enzyme of photosynthesis, originated in the Archean [79], long before the diversification of cyanobacteria [80] and the development of oxygenic photosynthesis as estimated by molecular phylogenies [71,81–83]. The O_2_-evolving complex evolved even before PSII in Terrabacteria chlorophototrophy ([84]).

A low oxygen concentration is needed for steroid synthesis and aerobe metabolism [85–87]. Based on experiments on living microbes, the free-living α-proteobacterial ancestor of the mitochondrion could have required only 3 nM–1 μM O_2_ (about 0.001−0.4% PAL -Present Atmospheric Level-O_2_) to respire aerobically and sterol synthesis in yeast is possible under oxygen concentrations as low as 7 nM O_2_ (0.003% PAL O_2_) [42].

Taken together, these observations imply that sufficient concentration of molecular oxygen was available early on (whether abiotic or biological in origin) and was not a limiting factor for eukaryogenesis. The time constraint for eukaryogenesis is more biological. Stromatolites from the Strelley Pool Formation (Australia) that suggest photosynthesis and include organic matter with isotopic signatures of carbon, sulfur and nitrogen metabolisms, indicate that the minimum age of the domain Bacteria is probably more than 3,42 Ga ([88]; reviews in [89,90]). The probable minimum age of the domain Archaea is based on strongly ^13^C-depleted isotopic composition of organic-rich lacustrine shales from the 3 Ga Lalla Rookh sandstone Formation, Australia, indicating methanogenesis but also possibly acetogenesis by bacteria [90,91] and in 2.7 Ga Tumbiana stromatolites, Australia [92]. Molecular clocks estimates place the origin of the domains Bacteria and Archaea in the Archean or the late Hadean [93]. The ages of the last common ancestors of the Asgard archaeon and the alphaproteobacterium involved in eukaryogenesis are estimated by a recent molecular clock at, respectively, 2.67−2.19 Ga for the nuclear archaeal lineage and 2.58−2.12 Ga for the mitochondrial bacterial lineage, while LECA is inferred to have originated 1.93−1.84 Ga and plastids diverged from free-living Cyanobacteria 2.14−1.73 Ga [94]. These estimates are challenging and may vary with the variable rate of molecular evolution, selected fossil calibrations (and their rarity and debated ages and identities, in some cases), as well as the root maximum age and position selected to calibrate clock models [18,94]. This explains the older estimate of LECA between 2.386 and 1.958 Ga by Strassert *et al*. [18], implying older mitochondrial and nuclear FECAs.

While a range of redox proxies can be used to reconstruct palaeoredox conditions in ancient sedimentary successions, they lack time and spatial resolution and accuracy, and they can be altered [71,95,96]. Moreover, in early microoxic niches, it is possible that the molecular oxygen source and sink were balanced so that no accumulation could be preserved and detected [95]. Indeed, today, microbial respiration of O_2_ at nanomolar concentrations is ubiquitous and drives microbial activity in seemingly anoxic aquatic habitats [76]. This would have been the case for early eukaryotes living in micro- or nanooxic niches. Microfossil assemblages are also subject to preservation and sampling biases, and they represent ecological communities mixed in time (depending on sedimentation rate and size of studied fossiliferous rock sample) and space (vertically—depending on water column and sediment depth, and laterally—depending on possible transport). Combining redox proxies and microfossil analyses at high resolution along palaeoenvironmental gradients can provide some hints to the palaeoecology of early eukaryotes and in some cases may suggest that some early eukaryotes thrived in anoxic or microoxic niches [97,98], as several modern eukaryotes do today. However, although presumably enough oxygen was available in non-sulfidic areas of oceanic and perhaps terrestrial habitats close to cyanobacterial mats through the Proterozoic and even earlier in the Archean, the very low oxygen concentration sufficient for steroid synthesis and aerobe metabolism is undetectable by most geochemical proxies. Recently the presence of a particular microfossil taxon was proposed as a possible direct palaeoredox proxy. *Navifusa majensis* is an elongate oval-shaped smooth-walled microfossil with rounded ends that is known in Palaeoproterozoic to Neoproterozoic fossil assemblages, although other species of the genus are reported in the Palaeozoic [99]. The ultrastructural analysis of 1 Ga and 1.75 Ga specimens revealed the exquisite preservation of internal membranes interpreted as thylakoids based on their size, fine structure and arrangement [95]. The presence of fossil thylakoids—the membranes where oxygenic photosynthesis takes place—permitted the unambiguous identification of *Navifusa* as a cyanobacterium capable of performing oxygenic photosynthesis and perhaps also anoxygenic photosynthesis, as some modern cyanobacteria are capable of both metabolisms and can easily switch between the two, depending on diurnal variations in local light, H_2_S concentration and nutrient availability in microbial mats [100]. Recently, inner membranes also interpreted as thylakoids were reported in silicified cyanobacteria from the 0.76 Ga Draken Formation, Spitsbergen [101].

### (d) The Palaeoproterozoic context

The Palaeoproterozoic (2.5 to 1.6 Ga) was a time of great changes in early Earth evolution, starting with the 2.5−2.4 to 2.1−2.0 Ga transition to a permanent planetary increase in atmospheric oxygen during the GOE [66,67,69,102], the extensive Huronian glaciations between 2.45 and 2.2 Ga [103], the enigmatic 2.3−2.1 Ga Lamagundi-Jatuli carbon isotopic anomaly [104], the 2.45 Ga break-up of the Neoarchean supercontinent Kenorland [105] and the 1.85 Ga assembly of the supercontinent Nuna-Columbia [106], as well as variations in nutrient availability and heterogenous redox conditions of marine environments [102,107,108]. In the late Palaeoproterozoic to early Neoproterozoic (1.8 Ga to 0.8 Ga), the global climate conditions became more stable, with a balanced rock cycle and no glaciations [70,109,110], stable Earth rotation speed and Earth–moon tidal torques with a stable day length (19 h) [111], a stable carbon cycle with little carbon isotopic fluctuations [110,112,113] and balanced mantle convection despite tectonic events such as the formation and fragmentation of the supercontinent Rodinia between 1.2 and 0.7 Ga. However, redox conditions were heterogenous in oceanic basins. This billion-years-long stability is proposed to be called the ‘balanced billion’ [114] to replace the former subjective ‘boring billion’ appellation. Following this stable period of the early Neoproterozoic (Tonian, 1.0−0.8 Ga) came a time of great variations, with another global increase in oxygenation (0.8–0.6 Ga Neoproterozoic Oxidation Event (NOE)) and the global glaciations of the Cryogenian (720–635 Ma), followed by the Ediacaran period [102].

From the palaeobiological point of view, the ‘boring’ or ‘balanced’ billion was actually an ‘exciting’ billion for the evolution of life [115,116]. The unambiguous Palaeoproterozoic microfossil record includes iron-loving and other undetermined filamentous and coccoidal prokaryotes, eukaryotes and cyanobacteria (for a review, see [115]). The body fossil record of eukaryotes includes unidentified protists [99,115,117–125] and multicellular fossils interpreted as debated algae ([54], but see [126,127]) or possible algae [55], as well as debated pyritized macrostructures [128], while the molecular record of protosterols is interpreted as possible evidence for stem eukaryotes and suggesting a late origin of LECA ([129]; but see [130]). Molecular phylogenies [4,8,18,94,131] and some palaeobiologists [118,119,124,132–134] proposed an early origin of eukaryotes in the Palaeoproterozoic, putatively in the Archean [115,135–139], and an early diversification of stem and crown eukaryotes [55,115,119,132,133,139], while others consider the body and molecular fossil record compatible with a younger late Mesoproterozoic LECA [129,140–142].

The currently known fossil record of unambiguous early eukaryotes started during the ‘balanced billion’. Older successions such as the 3.22 Ga Moodies Group, South Africa [136], the 3 Ga Farrel quartzite, Australia (EJ. Javaux personal observation, 2010), the 2.09−1.96 Ma Hutuo Group, China [143], the 2.1 Ga Francevillian Gp, Gabon [144] and the 1.9 Ga Kondopoga Fm, Karelia, Russia [145] preserve smooth-walled vesicles (leiospheres) that could be eukaryotic or prokaryotic. The 1634 ± 9 Ma Chuanlingguo Formation (Changcheng Gp) that preserves some of the oldest reported unambiguous eukaryotes (prior to this study) [99] was deposited in a redox-stratified ocean with fluctuating deeper water oxygenation [108]. The highly fluctuating redox conditions are proposed to have constrained the radiation of eukaryotes owing to oxygen and iron limitation [146] or to have restricted the ecological impact of photosynthesizing eukaryotes by limiting the bioavailability of micro-nutrients (trace metals essential in enzymes) [107], nitrogen [147] and phosphorus [148], thereby impacting plankton size and trophic complexity in oceans [149]. Based on biomarker data, the (late) rise of planktonic microalgae might have triggered the diversification of metazoans in the Ediacaran [150]. The Palaeoproterozoic eukaryotic record shows early moderate diversity and morphological disparity that are relatively similar to the following Mesoproterozoic and pre-cryogenian Neoproterozoic period and implies a deeper history of the domain Eucarya [122]. Previous studies [121,151–154] have already underlined the billion-year long delay between the oldest known Palaeoproterozoic eukaryotic fossils and the mid-Neoproterozoic eukaryotic diversity and ecological importance, also suggested by the biomarker record [129], although an increase in diversity occurred earlier in the early Mesoproterozoic [155]. A recent quantitative review of Proterozoic eukaryotic diversity also deduced a slow rise and relatively stable diversity with small-scale fluctuations during the approximately 1.8–0.8 Ga ‘balanced billion’, contrasting with a rapid and dynamic increase of taxonomic richness and several radiations followed by extinctions of large unicellular acanthomorph (spiny) microfossils (the ‘DPA’ Doushantuo-Pertataka acritarchs) possibly caused by cooling during the Cryogenian, and of the Ediacaran metazoan biota owing to fluctuations in ocean oxygenation in the Ediacaran [123]. By contrast, an ecosystem model proposed that the size distribution of preserved eukaryotic microfossils from approximately 1.7 Ga and onward required an active eukaryote ecosystem of large biomass, with various metabolisms and modes of feeding, and with eukaryotic algae contributing to about half of total marine primary production [156].

The oldest commonly accepted fossils of crown-group eukaryotes include the 1.05 Ga red alga *Bangiomorpha pubescens* [51]; the 0.95 Ga green alga *Proterocladus antiquus* [52]; the 1.03 Ga probable alga *Arctacellularia tetragonala* [53]; the 1.01−0.89 Ga probable fungi *Ourasphaira giraldae* [157] and 0.75 Ga testate amoebae [158,159]. A recent observation of *Arctacellularia* specimens with a thin external sheath by C. Demoulin [160] may suggest some similarity to the cave branching cyanobacteria *Geitleria calcarean* [161], although these cyanobacteria have a broad calcified sheath enclosing a filament of smaller cells (5–10 µm in diameter and 7−20 µm in length) whereas *Arctacellularia* are larger (25–45 μm wide and 15−100 μm long cells) and show intercalary division rather than apical filament growth. Another bacterium, the sulfur-oxidizing *Thiomargarita magnifica,* can reach millimetric to centimetric cell size in filaments with asymmetric division [162] but is not branching or photosynthetic, unlike *Arctacellularia*, which preserves intracellular remains of chlorophyll [53]. Recently reported 1.64 Ga multicellular filamentous eukaryotes were interpreted as possible green algae [55] and future detailed analysis of their wall and internal ultrastructure and chemistry would strengthen this interpretation. These fossils provide minimum ages that can be used to calibrate molecular clocks and suggest an earlier origin of the supergroups Opisthokonta and Amoebozoa (Obazoa) and an even earlier origin of the Archaeplastida and of the primary plastid endosymbiosis—and consequently, of LECA. Considering that stem and crown-group eukaryotes existed during the ‘balanced billion’, the causes of (i) the delay in diversification of eukaryotic phenotypes (mostly documented based on fossil morphology, which might not reflect genotypic and ultrastructural diversity), (ii) their presumed late ecological importance (suggested based on the biomarker and microfossil record) and (iii) the increased post-cryogenian taxonomic morphological diversification (both of non-metazoan and metazoan microscopic and macroscopic eukaryotes) remain puzzling and unresolved despite possible environmental and biological constraints evoked above [118,119,123]. A combination of several biological and environmental factors might be involved at different times and for specific events rather than a global cause, so this global pattern should be examined in detail (for example, an increase in diversity had occurred already in the early Mesoproterozoic [155]) and refined with new discoveries of Proterozoic eukaryotic fossils. Recently, another reassessment of eukaryotic diversity from 1890 to 720 Ma concluded that the patterns of early eukaryote diversity reported in previous studies based on biomarkers and on body fossil data were actually an artifact of sampling and did not support a diversification in the Tonian [125]. Therefore, the pattern of early eukaryotic diversification remains obscure. In addition to the causes and timing of this diversification pattern, debates centre around the timing of eukaryogenesis and the age of LECA, the interpretation of early eukaryotic fossils and the diversification of crown clades, including the timing of the plastid primary endosymbiosis.

In this paper, I present new data from a 1.78 to 1.73 Ga fossil assemblage in the Palaeoproterozoic of Australia that preserves the oldest known eukaryotic microfossils to date, as well as cyanobacteria with preserved thylakoids [95], and discuss how cellular palaeobiology might provide some insight to the debates.

## 2. Material and methods

**Figure 1.**
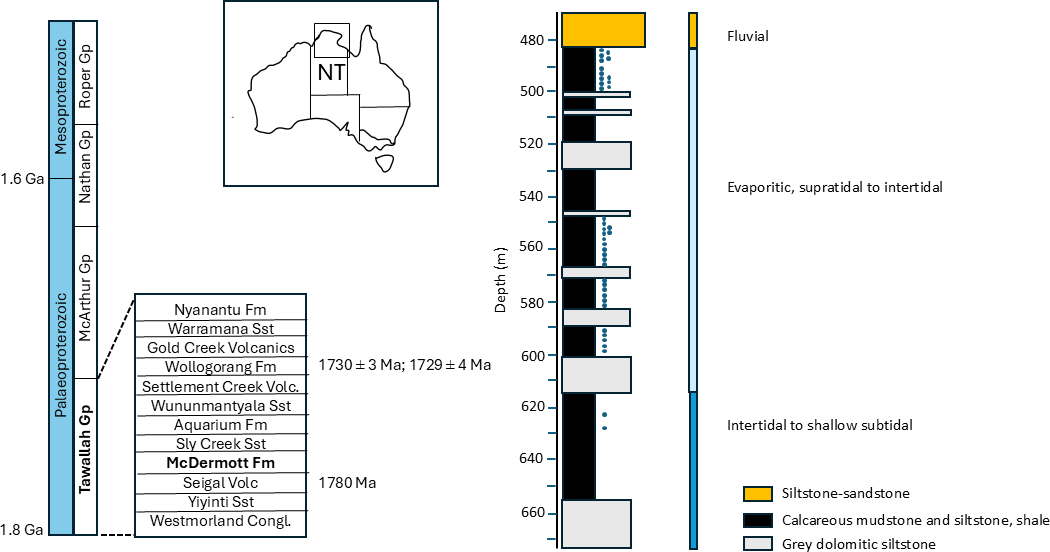
Simplified stratigraphy of the southern McArthur Basin in Northern Territory, Australia, and sedimentary log of the McDermott Formation in drill core GSD7. Ages of the Wollogorang Fm are SHRIMP U-Pb zircon ages from tuffaceous mudrocks [166] and for the Seigal Volcanics conformably underlying the McDermott Fm are from [167]. Dots mark the approximate position of samples in core GSD7. Log and environmental interpretation modified from Spinks *et al*. [163].

The McArthur Basin in northern Australia is an intracontinental Palaeoproterozoic–Mesoproterozoic (1.85−1.45 Ga) succession of non-metamorphosed marine, shallow-marine to fluvial sediments and interbedded volcanics [163]. The stratigraphy of the southern McArthur Basin, NW Australia, comprises the Tawallah, McArthur, Nathan and Roper groups [164]. The McDermott Formation, which is part of the ca 1790 to 1710 Ma Tawallah Group (also called the Redbank Package [165]), was sampled for this study (figure 1). The McDermott Formation consists of basal sandstone deposited in shallow-marine environment, interbedded carbonaceous siltstone and stromatolitic dolostone with carbonate pseudomorphs after gypsum deposited in mostly evaporitic environment, interbedded siltstone and mudstone, and upper fluvial and estuarine sandstones and red mudstones [163]. Based on trace element and mineralogical palaeoredox proxies [168], the McDermott Formation was deposited under mostly sulfide-limited suboxic to anoxic conditions, in an intracontinental near-shore marine setting. Fe speciation data suggest heterogenous conditions with oxic to equivocal conditions mostly in the supratidal to intertidal environments and equivocal to mostly ferruginous conditions in the intertidal to shallow subtidal environments but no euxinic conditions [169]. Short-lived and localized pyritic laminated shale beds, along with gypsum pseudomorphs and evaporite needles in the lower McDermott Formation, indicate some sulfate availability [163], but the absence of euxinia in overlying sediments that were deposited in a shallow-marine intracontinental near-shore environment contrasts with the presence of euxinic conditions in equivalent shallow environments in the open marine system around 1.8 Ga [170,171]. The depositional age of the McDermott Formation is constrained by an approximate minimum age of 1780 Ma of the conformably underlying Seigal Volcanics [167,168]. The McDermott Formation is overlain successively by the Sly Creek Sandstone, the Aquarium Formation, the Wununmantyala Sandstone, the Settlement Creek volcanics and the Wollogorang Formation. The Wollogorang Formation is dated by SHRIMP U-Pb zircon ages of 1730 ± 3 Ma and 1729 ± 4 Ma from tuffaceous mudrocks [166] and was an euxinic open-marine basin [163] with anoxia in the photic zone, consistent with the occurrence of biomarkers of strictly anaerobic photosynthetic green sulfur bacteria [172].

Horizons targeted for sampling included 42 samples of fine-grained grey mudstones and siltstones from one drill core through the McDermott Formation (drill core GSD7, drilled by BHP Minerals Pty Ltd in 1994 [163]). Forty samples are from the arid supratidal to intertidal marine facies and two from the transition to subtidal facies deeper in the core, as described in Spinks *et al*. [163] (figure 1). Drill core samples were requested and received from T. Spinks and M. Kunzmann at Darwin drill core facility, Australia. About 25 g of each sample was demineralized by treatment with HF/HCl followed by settling and decanting to retrieve fragile organic-walled microfossils. Part of the residue was mounted on microscope slides. Microfossil images are transmitted light micrographs, in some cases with differential interference contrast, using a Zeiss Axioimager microscope equipped with an Axiocam MRc5. Specimens are reposited at the Early Life Lab collection, University of Liège, Belgium, with England Finder coordinates and slide reference numbers indicated in figure captions. Specimens of *N. majensis* from the McDermott Formation were pipetted under an inverted microscope and prepared for resin embedding. Unstained ultra-thin sections were prepared as described in [95] and observed by TEM at the Microscopy CORE Lab, University of Maastricht, in the Netherlands.

## 3. Results

### (a) Description of fossil assemblage

Twenty of the 42 studied samples from the McDermott Formation are fossiliferous. The microfossils are organic-walled and preserved as carbonaceous compressions in fine-grained siltstones and mudstones. The McDermott microfossil assemblage includes a diverse collection of entities.

#### (i) Spheroidal and filamentous forms of undetermined possible prokaryotic or eukaryotic affinity

**Figure 2.**
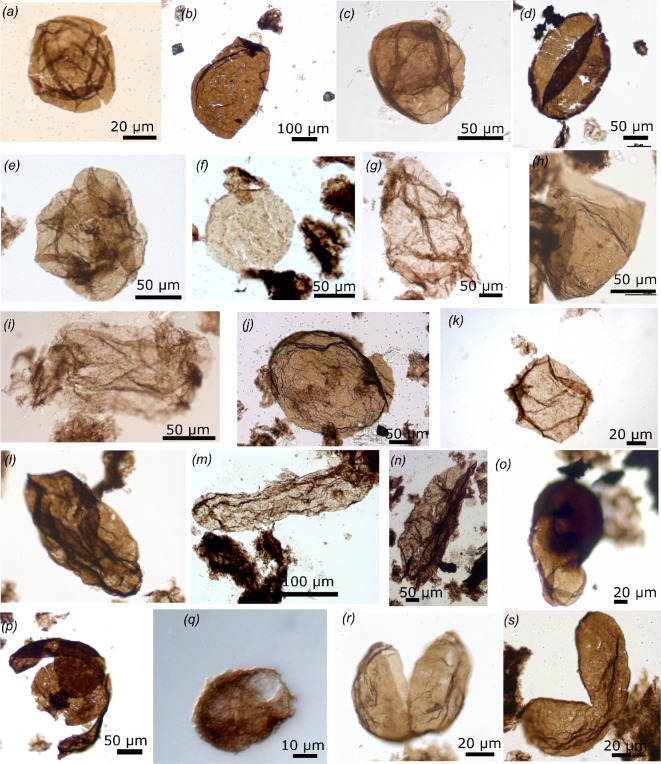
Smooth-walled vesicles (slide number, England finder position on slide, depth in core GSD7). (*a*) 75 471 *L. crassa* (623.8_624.1 m), (*b*) 75 473 *L.* sp., with a cork-like texture (623.8_624.1 m), (*c*) 75432 C34/4 *L. jacutica* (491.9_492.2 m), (*d*) 77549 H33 *L.* sp., with a cork-like texture (486.6 m), (*e*) 77555 J42 *L. jacutica* (487.3 m), (*f*) 75442 P32 *L.* sp., with granular aspect of the wall (493.9_494.3 m), (*g*) 75431X42/2 *L. tenuissima* (491.9_492.2 m), (*h*) 77554 T37 *L*. sp., large enrolled fragment with faint striations unlike the concentric striations of *Valeria lophostriata* (487.3 m), (*i*) 75432 T39/1 *L*. *tenuissima* (491.9_492.2 m), (*j*) 75438 J46/4 *L*. *tenuissima* (493.9_494.3 m), (*k*) 75431 Q40/3 *L.* sp., with granular aspect of the wall (491.9_492.2 m), (*l*) 77548 U43/3 *Schizofusa sinica* (486.6 m), (*m*) 75440 K45 *Cucumiforma* sp. (491.9_492.2 m), (*n*) 75442 P30/2 *L.* sp.*,* large oval vesicle (493.9_494.3 m), (*o*) 75473 B39/4 unnamed form A, smooth-walled vesicle with lanceolate folds, a deep constriction separating two sides of different sizes and with an ICI in one side (623.8_624.1 m), (*p*) 77554 K37/2 smooth-walled vesicle with an opening along four curved slits (487.3 m), (*q*) 79272 B39 *L. kulginica,* with a fluffy wall texture and a pylome (494.9_495.3 m), (*r*) 75471 Z41/2 *L. crassa,* opening by medial split (623.8_624.1 m), (*s*) 77555 G33/3 *L.* sp., with a brown cork-like texture, opening by medial split (487.3 m).

Large (up to a few hundred microns in diameter) smooth-walled spheroidal to oval vesicles with different sizes, colours and textures can be abundant in most fossiliferous samples. They include rare *Cucumiforma* sp. (figure 2*m*), an oval-shaped (321.4 µm long and 64.4 µm wide) vesicle with rounded ends and longitudinal discontinuous folds oriented from pole to pole [99], considered likely eukaryotic [123] or possibly eukaryotic [125]. Abundant spheroidal vesicles include *Leiosphaeridia crassa* (<70 µm in diameter) (figure 2*a,r*) and *L. jacutica* (>70 µm up to 433.6 µm in minimum diameter) with thick lanceolate folds (figure 2*c,e*)*, L. minutissima* (<70 µm) and *L. tenuissima* (>70 µm up to 320 µm) with thin sinuous folds (figure 2*g*,*i,j*)*,* identified as in [133], and unnamed *Leiosphaeridia* spp. with different (cork-like, faintly striated, granular) wall textures (figure 2*b*,*h,k*) including very large (more than 173 µm wide and 555 µm long) oval vesicles with fine folds (*L.* sp.) (figure 2*n*). Rare specimens of fusiform vesicle (98.48 µm wide and 76.3 µm long) displaying a slit-like opening with bordered folds extending from pole to pole (*Schizofusa sinica; *figure 2*l*) also occur, and are considered likely eukaryotic [125]. Unnamed form A (figure 2*o*) consists of a medium brown shagreenate vesicle (180.31 µm long) with a deep invagination between two (45.78 and 74.25 µm wide) rounded sides and an intracellular inclusion (ICI) present within the larger side of the vesicle. These entities are of uncertain prokaryotic or eukaryotic affinity because of their simple morphology that is known in both prokaryotic and eukaryotic clades. Surprisingly, colonies of smooth-walled vesicles (*Synsphaeridium*) that are usually diverse and common in Proterozoic assemblages are not observed.

**Figure 3.**
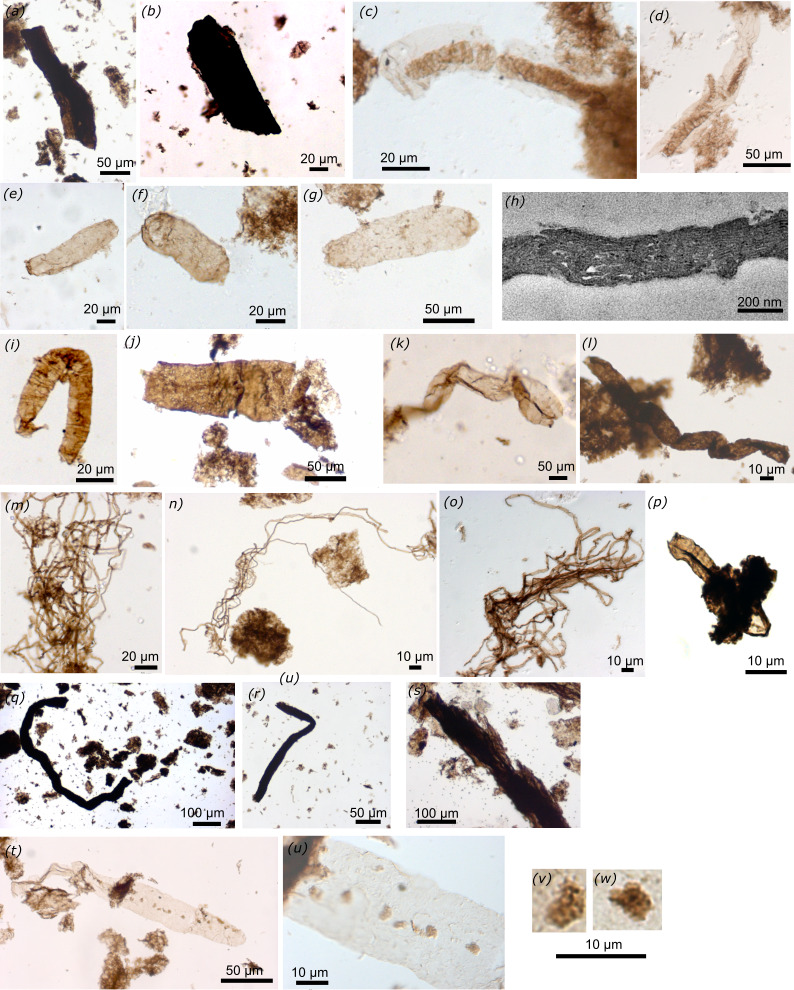
Probable and unambiguous cyanobacteria and other filamentous taxa (slide number, England finder position on slide, depth in core GSD7). (*a*) 75464 L43/4 *Changchengonema densa,* dark brown tube fragment with sheath (596.5_596.8 m), (*b*) 75464 S58 *C. densa,* dark brown tube fragment with sheath (596.5_596.8 m), (*c*) 75431 *Paleolyngbya barghoorniana,* sheathed unbranched uniseriate filament with discoidal cells and transparent sheath (491.9_492.2 m), (*d*) 75440 G47 pseudobranching filament with discoidal cells and transparent sheath (491.9−492.2m), (*e*) 75431 *N. majensis* (491.9_492.2 m), (*f*) 75431 D31 *N. majensis* (491.9_492.2 m), (*g*) 75431 J27/1 *N. majensis* (491.9_492.2 m), (*h*) TEM image showing thylakoidal membranes preserved within a transversal ultra-thin section of *N. majensis,* (*i*) 75438 Z42 *Rugosoopsis tenuis* (493.9_494.3 m), (*j*) 75464 M35 *Siphonophycus gigas* (596.5_596.8 m), (*k*) 75442 Y57 *Siphonophycus kestron* (493.9_494.3 m), (*l*) 75463 *Siphonophycus solidum* (596.5_596.8 m), (*m*) 75437 S56/3 *Siphonophycus robustum,* bundle of 2 µm wide filamentous sheaths (493.9_494.3 m), (*n*) 77555 R48/1 *Siphonophycus septatum,* bundle of 1 and 2 µm wide filamentous sheaths (487.3 m), (o) 75431 *Siphonophycus typicum,* bundle of 5 µm wide filamentous sheaths (491.9_492.2 m), (*p*) 79271 K48/1 *Tortunema wernadaskii,* unbranched filamentous sheath with annular imprints (peudoseptate filament) (494.9_495.3 m), (*q*) 75463 N44 long dark brown tube fragment (596.5_596.8 m), (*r*) 75465 E61/3 long dark brown tube fragment (596.5_596.8 m), (*s*) 75442 P31/1 large fibrous tubes and sheath (493.9_494.3 m), (*t,u*) 75439 X44 groups of small dark brown vesicles distributed irregularly on the surface of a light brown specimen of *N. majensis*, and interpreted as possible ectobionts (493.9_494.3 m), (*v,w*) magnification of the possible ectobionts.

#### (ii) Probable cyanobacteria

Abundant bundles of entangled filamentous sheaths ripped from microbial mats are of probable cyanobacterial origin and include several species of *Siphonophycus* spp. (figure 3*j–o*) that are differentiated based on their diameter, as in [173]: *Siphonophycus septatum* (1–2 μm), *S. robustum* (2–4 μm)*, S. typicum* (4–8 μm), *S. kestron* (8–16 μm in width, more than 160 µm long), *S. gigas* (17–73.95 µm in diameter dark brown tube and up to 170.61 µm long fragment). Two specimens of *Paleolyngbya barghoorniana* (figure 3*c*) (3.77–7 µm wide) filaments with uniseriate discoidal cells (1.87 µm in length and 17.83 to 21 µm in width) in a (115 µm long) transparent sheath*,* interpreted as a probable hormogonian oscillatoriocean cyanobacterium [174], and one pseudo-branching filament with uniseriate (6.13 µm in width and 3 to 4 µm long) discoidal cells in a (20.5–29 µm) wide sheath of probable Oscillatoriale cyanobacteria (figure 3*d*) also occur. One 66 µm long fragment of unbranched filamentous sheath with annular imprints (peudoseptate filament) is identified as *Tortunema wernadaskii* (figure 3*p*) and two specimens of a (12.72 µm wide and 111.78 µm long) filamentous sheath with transversal folds or fabric are identified as *Rugoopsis tenuis* (figure 3*i*). These filamentous microfossils are usually interpreted as cyanobacteria based on their morphology, size and habitat and comparison with modern taxa and further analyses of their chemistry (for example the presence of unique pigments [175]) and ultrastructure would strengthen this interpretation [174].

**Figure 4.**
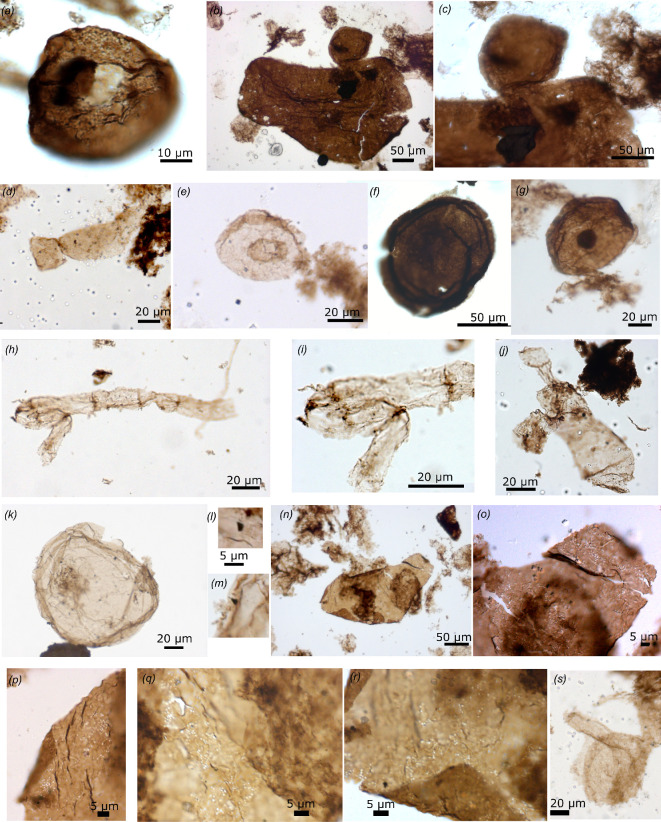
Possible and unambiguous eukaryotes (slide number, England finder position on slide, depth in core GSD7). (*a*) 75431 S51 1/2 *Dictyosphaera macroreticulata*, vesicle with a wall made of organic plates and opening by a pylome with its attached lid (491.9_492.2 m), (*b*) 75434 T48 1/2 *Gemmuloides doncooki,* shagrinate vesicle with a bud, (*c*) magnification of smaller budding vesicle (492.9_493.3 m), (*d*) 75438 P29/4 *Jacutianema solubila,* morphotype with two attached cylindrical vesicles of different size (493.9_494.3 m), (*e*) 75438 U31 *Pterospersimorphita* sp., central translucent vesicle in vesicle construction (493.9_494.3 m), (*f*) 75471 R54 *Simia annulare,* vesicle with a narrow ripped translucent flange visible at the periphery (623.8_624.1 m), (*g*) 75438 U41 *S. annulare,* vesicle with a narrow translucent flange at the periphery and an ICI (493.9_494.3 m), (*h*) 75465 K59 *Siphonoseptum* sp.*,* uniseriate unsheathed filament with a thin wall and dark septa ornamented by ruffles between cells that are longer than wide and of different lengths; (*i*) magnification of ruffles (sinuous filaments) attached at septa (596.5_596.8 m), (*j*) 75 442 U40/4 cf. *Siphonoseptum* sp., barrel to oval-shaped cells with a slight central constriction at possible septum, a lateral filamentous expansion on one side, and a tubular expansion at one end separated by a septum from a terminal spheroidal vesicle (493.9_494.3 m), (*k*) 75473 B42/4 unnamed form B, vesicle ornamented with a few spiny dark conical processes, irregularly distributed on the vesicle wall (623.8_624.1 m), (*l,m*) magnification of conical spines of unnamed form B (*o–r*) 75437 E38/3 fragment of wall perforated by oval holes interpreted as possible traces of eukaryovory, possiby by myzocytosis (protist vampirism) (493.9_494.3 m), (*s*) 75438 X33/4 unnamed form C, vesicle with a tubular expansion and a partial sheath (493.9_494.3 m).

#### (iii) Unambiguous cyanobacteria

Unequivocal cyanobacteria occur as abundant (more than 200 specimens in some samples) oval-shaped (4.1–40.1 µm in width and 26–176.81 µm in length, *n* = 28) *Navifusa majensis* (figure 3*e–h,t,u*). A recent ultrastructural study of *N. majensis* specimens from the McDermott Formation revealed exquisitely preserved internal membranes interpreted as thylakoids (figure 3*h*) based on their size, distribution and ultrastructure, directly demonstrating the presence of cyanobacteria able to perform oxygenic photosynthesis [95]. As mentioned above, some cyanobacteria switch easily between oxygenic and anoxygenic photosynthesis in sulfidic environments [100]. This versatility could have been more prevalent in Palaeoproterozoic sulfidic open oceans [176] but perhaps less in intracontinental basins protected from euxinia where the McDermott Formation studied here was deposited, as suggested by palaeoredox analyses [163,169]. The simple morphology of this fossil is also known in other prokaryotic or eukaryotic clades so its cyanobacterial identity in other successions requires confirmation with ultrastructural and/or chemical analyses, as performed for specimens of the 1.78-1.73 Ga McDermott Formation and the 1.0−0.9 Ga Shaler Group [95]. It is difficult to assess the benthic or planktonic habit of *Navifusa*, although it resembles the modern benthic oval-shaped *Cyanothece* [177].

#### (iv) Possible and unambiguous eukaryotes

One specimen of *Dictyosphaera macroreticulata* (figure 4*a*; 42.2 µm in diameter) has a wall made of small (0.7−0.8 µm) polygonal organic plates and shows a pylome, a circular cyst opening structure of 10 µm. An unnamed vesicle (102.42 µm in diameter) bears small conical dark spines (1.5 µm wide and 2.5 µm long) (unnamed form B, figure 4*k*–*m*). These microfossils are interpreted as eukaryotes based on their complex morphology. The synthesis and assembly of imbricated organic plates making up *Dictyosphaera*’s wall suggest the presence of a complex cytoskeleton and endomembrane system [117,178–180]. One large (224.73 µm wide and 406 µm long) somewhat oval vesicle with a shagreenate wall and with one attached smaller (99.5 µm wide) spheroid or bud is attributed to the *Gemmuloides doncookii* (figure 4*b,c*). Budding is not a reproduction mode unique to eukaryotes but here it is combined with a large vesicle size and ornamented cell wall, implying an eukaryotic identity for this taxon.

Other taxa comprising multicellular entities include: (i) a 66.55 µm vesicle with a 15.72 µm wide and 98.21 µm long tubular expansion and a partial sheath (unnamed form C, figure 4*s*); (ii) two specimens consisting of one elongate vesicle attached to a spheroidal vesicle with a deep constriction between them (87.98 µm long to 30 µm wide vesicle attached to a 26.17 long to 28 µm wide spheroid, and a 43.09 µm long and 24.95 µm wide oval vesicle linked to 32.62 long to 14.87 µm wide vesicle), different from barrel-shaped cells with terminal lanceolate folds of *Arctacellularia* and resembling *Jacutianema solubila* (figure 4*d*). These multicellular microfossils are probable eukaryotes based on the combination of unusual multicellular morphology and large cellular size, and are left in open nomenclature owing to their rarity, preventing a complete understanding of their construction.

Brown shagreenate (67–104 µm in diameter) vesicles identified as *Simia annulare* surrounded by the remnants of a narrow (3 to 8.3 µm wide) lighter translucent membrane with a sharp boundary from the vesicle wall are usually interpreted as ornamented protists with a narrow equatorial flange (figure 4*f,g**, n* = 3, one specimen with an intracellular inclusion-ICI) or possible eukaryotes [123]. This spatial organization (whether it is a flange or an external membrane) can be difficult to assess in flattened specimens although the position of the flange at the periphery differs from the vesicle-in-vesicle construction of *Pterospersimorphita* where the enclosed vesicle occupies a central position away from the external vesicle wall [181]. Recently Silurian specimens of *Simia* sp. were interpreted as euglenids based on their ultrastructure [182], and this interpretation was extrapolated to 1.09 Ga Nonesuch Fm specimens [93] although no ultrastructural analyses was performed on those. However, these *Simia* sp. show several circular bands forming the vesicle and differ from *S. annulare*, which has a narrow translucent flange around the vesicle delimited by a sharp wall boundary, also reported in many Proterozoic successions such as the Palaeoproterozoic Changcheng Gp in China [55] or the 1.1 Ga El Mreiti Gp in Mauritania [183].

Rare *Pterospersimorphita* sp. (figure 4*e*) that consist on large vesicles enclosing a smaller translucent central vesicle (disphaeromorph, 39.25–52.5 µm diameter outer vesicle enclosing a 12.35–23.92 µm inner vesicle) are interpreted as possible protists. The eukaryotic nature of *Pterospersimorphita* spp. was recently questioned [122,124], while considered possibly eukaryotic by Tang *et al.* [123] and Porter *et al.* [125]. Indeed, this genus includes a variety of spheres enclosing spheres with variable size and opacity. Specimens where the internal vesicle is opaque are ambiguous as this dark inclusion could correspond to a collapsed cytoplasm (ICI) rather than a real vesicle. However, to my knowledge, the construction of a large cell enclosing another single translucent central large cell is not known in prokaryotes, which can have several small cells in a common envelope (e.g. the colonial cyanobacteria *Gloeocapsa* [175] or the pleurocapsale *Staniera* cell wall enclosing small cells called baeocytes [177,184]).

A fragment of multicellular unsheathed thin-walled filament of about 200 µm long and 12.43–14 µm in diameter, with six barrel-shaped cells of various lengths (28.67 to 44.67 µm long) and separated by dark septae but with no constrictions between the cells resembles *Siphonoseptum bombycinum* (figure 4*h,i*) [122]. This single fragment has sinuous filaments (‘ruffles’) hanging from septae, but it does not show the occasional dark spheres at septae observed by Riedman *et al.* [122]. Although the diameter of this fragment is smaller than *S. bombycinum* (21–92 µm in width) and overlaps with *Oscillatoriopsis* (1–25 µm), it differs from this unsheathed cyanobacterial filament that has cell length smaller than their width [173] and is tentatively identified as *Siphonoseptum* sp. Another specimen comprising a 85.25 µm long and 38 µm wide barrel- to oval-shaped cells with a slight constriction, with a side folded expansion and a 15.85 µm long tube separated by a septum from a 13.87 µm wide terminal sphere, might also correspond to a deformed larger fragment of *Siphonoseptum* or to an unnamed multicellular taxon (cf. *Siphonoseptum*?, figure 4*j*). These specimens also differ from the filamentous branching alga *Arctacellularia* that has strong constrictions between barrel-shaped cells and a larger diameter [53], and is sometimes envelopped by a thin sheath [160]. The possible alga *Qingshania* from the 1.64 Ga Chuanlinguo Fm in north China [55] also shows a similar morphology although it has a thicker wall and larger size, no ruffles, but intracellular spheroids (although these are extremely rare [55]) and rounded terminal cells. *Siphonoseptum* is interpreted as eukaryotic [122] based on the presence of occasional internal spheres not observed here, and characters observed here such a rounded terminal cell, some ornamentation or side expansion at septae, as well as the absence of sheath and the size, the barrel shape of the cells and the absence of strong constrictions between cells. Note that in some cyanobacteria, the presence or absence of a sheath can be a taxonomic trait or an ecological trait and some unsheathed filamentous cyanobacteria have cells longer than wide, of various sizes (cells rarely isodiametric) and without constrictions at septa, such as *Geitlerinema*, or *Limnothrix*, although their diameter is smaller (2 to 7× longer than wide, up to 14 µm long) [185] and they do not show the combination of other traits observed in *Siphonoseptum* that suggests an eukaryotic affinity.

#### (v) *Tappania plana*, an unambiguous protist

**Figure 5.**
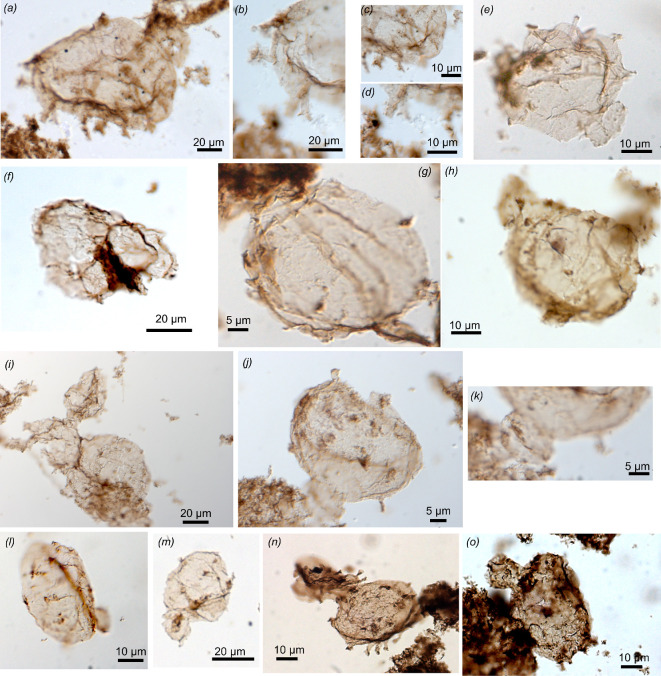
*Tappania plana* (slide number, England finder position on slide, depth in core GSD7). (*a*) 75431 D32 vesicle with neck-like expansion and tubular processes (491.9_492.2 m), (*b*) magnification to show short evased tubular end with dark rim and (*c,d*), straight tubular processes, (*e*) 75466 Y29 vesicle with neck-like expansion, tubular processes arising from bulbous protrusions and with dark closed ends (596.5_596.8 m), (*f*) 75439 X40 vesicle with neck-like expansion (491.9_492.2 m), (*g*) 75464 N44/2 vesicle with short processes and a long expansion (596.5_596.8 m), (*h*) 75464 S38/3 vesicle with one neck-like expansion, one large process and small closed processes with dark ends (596.5_596.8 m), (*i*) 75464 J33 four attached vesicles, one attached on the side by a neck-like expansion, vesicles with tubular processes, conical processes (596.5_596.8 m), (*j*) 77465 L 59 vesicle with an open neck-like expansion, a closed neck expansion and processes (596.5_596.8 m), (*k*) magnification of the open extremity of a neck-like expansion (596.5_596.8 m), (*l*) 75466 A46 vesicle with one neck-like expansion and small darker spots that might be future processes (596.5_596.8 m), (*m*) 75466 U33/1 vesicle with one bulbous expansion and small darker spots that might be future processes (596.5_596.8 m), (*n*) 75463 K32 two attached vesicles with tubular processes and conical spines, one vesicle folded (596.5_596.8 m), (*o*) 75463 U32/S32 vesicle with a bulbous protrusion and small processes (596.5_596.8 m).

The iconic protist *Tappania plana* (figure 5*a–o*) is common in some McDermott samples that also include abundant cyanobacteria *N. majensis*. It shows a variable dynamic plastic morphology suggestive of a vegetative cell, with irregular ovoid vesicles bearing up to six or more irregularly distributed heteromorphic processes that can be tubular or conical or flared or tubular, but arising from conical bulges from the vesicle and with an open end or an end closed with a dark plug. Vesicles may also bear up to three diagnostic trapezoidal neck-like extensions, and sometimes up to two or more bulbous expansions from the vesicle wall and communicating with the vesicle interior, suggestive of budding. In addition, one specimen consists of four attached process-bearing vesicles (figure 5*i*) with three vesicles joined by their vesicle wall and the fourth vesicle attached by a neck-like expansion, perhaps in a division stage or colony, different from previously reported specimens with two vesicles in binary division [186]. Another specimen consists of two attached vesicles (figure 5*n*), each with tubular processes and one with dark spiny conical processes. These specimens evidence simple multicellularity in *Tappania*, a character that was also previously documented by septate processes in other successions [124,133]. In another specimen, a neck expansion is open (figure 5*j,k*), as previously observed in one specimen of the younger approximately 1.45 Ga Roper Group, Australia [133] and in the 1.64 Ga Chuanlingguo Fm, China ([124], see also for a summary of morphological features and stratigraphic and geographic occurrences of *T. plana*). In younger populations, an external membrane is sometimes preserved [187] but has not been observed here. Despite its wide morphological variability, there are no obvious modes in size or form in *T. plana,* which does represent a single biological species [187]. The vesicle size (24 specimens observed, *n* = 19) ranges from 17.80–74.25 µm in width (mean: 32 µm) to 28.34–180.31 µm in length (mean: 47.42 µm); with processes ranging from 1.46–8.66 µm in width and 2.73–10.41 µm in length; trapezoidal neck-like expansion with a base ranging from 3.91–16.34 µm in width and 3.01–9.93 µm in length and broadening top up to 17.56 µm in width; and bulbous expansions ranging from 8.97–4.14 µm in width and 3.44–15.47 µm in length.

Long filamentous branching protrusions were recently reported in a few hundred nanometers cells of two Asgard archaea of the order Lokiarcheales [27,28] and in two new Hodoarcheales [30] . Another (uncultivated) Hodarchaeale has an elongated cell body of 1.5–to 5.2 μm long and 1.1−0.8 μm wide, prolonged by a rounded expansion at one pole [29]. Although informative, these characters of modern complex Asgard archaea do not necessarily represent ancestral features after more than 2 billion years of evolution from the closest archaeal ancestor of eukaryotes [10]. Moreover, as mentioned above, these archaea have simple cellular internal structure–with no endomembranes like most prokaryotic cells, and an actin cytoskeleton only in protrusions used for syntrophy and storage and not involved in division, suggesting a major difference from a eukaryotic cell organization [30]. The complexity shown by these archaea or by PVC bacteria that can form protrusions or warts (tubercles) and have internal membranes and other eukaryotic-like features [22] differs from the complex and plastic morphology and size of *Tappania. Tappania* is one or two orders of magnitude larger and bears complex structures such as neck-like expansions that can be open, heteromorphic appendices with various endings and sometimes branching and/or sometimes septate and multicellular. *Tappania* can also form multicellular associations of up to four ornamented vesicles. To the best of my knowledge, these features are not known in prokaryotes. The complex and flexible morphology of *Tappania* implies the presence of a complex cytoskeleton and endomembrane system of eukaryotic grade. Its tubular processes might have been used for osmotrophy to absorb dissolved organic molecules [133,178], as many protists can do [188], or perhaps for predation by myzocytosis or cellular vampirism, piercing another cell surface and sucking out its contents as food, as Provora and some other protists do [65,189,190]. *Tappania* could also have been a mixotroph, using photosynthesis and heterotrophy, but evidence for photosynthesis is so far lacking. *Tappania plana* is known in other younger late Palaeoproterozoic and early Mesoproterozoic successions (see [124] for a review) and possibly in late Mesoproterozoic successions where a few specimens with a neck-like expansion but no processes have been observed [97,155,191]. Its occurrence in the McDermott assemblage moves back its stratigraphic record by more than 100 Ma, making it the oldest eukaryote reported to date, with other McDermott protists. *Tappania* has been previously considered as a fungi; a crown-group eukaryote [187], or a possible stem eukaryote before LECA or stem of crown group after LECA [133], or a total group eukaryote [142].

#### (vi) Cyst opening structures

Common cyst opening structures in the form of medial split in smooth-walled vesicles (*Leiosphaeridia* spp.) with detached or attached halves imply the existence of a life cycle that includes a metabolically active vegetative stage and a resting cyst stage (figure 2*r,s*). Such opening is known in fossil and modern protists but also in some pleurocapsale cyanobacteria (*Staniera*) that open to liberate small cells called baeocytes [177,184] and is thus not unique to eukaryotes, although these cyanobacteria have diameters of up to 50 µm, so perhaps larger leiosphaerid vesicles with medial split could be eukaryotic. A pylome—a circular cyst opening—of 12.4 µm is present in a specimen of *Leiosphaeridia kulgunica* with a 35.25 µm diameter and fluffy wall texture (figure 2*q*). A circular pylome is also present in *D. macroreticulata* as described above (figure 4*a*). A rare and unusual opening structure observed in one smooth-walled vesicle of 176 µm diameter operates along four curved slits, opening the vesicle like a flower (figure 2*p*). An opening at the end of a neck-like expansion of *T. plana*, a processes-bearing protist, is interpreted as a cyst opening structure (figure 5*j,k*) and has been previously observed also in one specimen from the 1.4 Ga Roper Group, Australia [133,178] and in the 1.64 Chuanlingguo Fm, China [124]. These excystment structures are more sophisticated than medial splits and require a sophisticated cytological control considered of eukaryotic grade.

#### (vii) Possible direct evidence for symbiosis

A fragment of a large sheath or cell wall (100 µm wide and 226.25 µm long) is perforated by numerous irregularly distributed (0.46 to 0.84 µm) oval holes with net borders, which suggest the possible presence of micropredators (figure 4*o–r*). Such holes have been observed in younger Proterozoic and Palaeozoic smooth-walled and ornamented microfossils, with sizes ranging generally from 0.1 to 9 µm (one example up to 16 µm) in diameter but unrelated to vesicle size (review in [192]). They seem unrelated to mineral perforations which are irregular or polygonal and often tier the organic wall, and to vesicle ornamentation, which should be present on all specimens of a same taxon. The regular shape and size of the perforations on a single specimen also differ from microbial decay caused by a consortium of microbes that degrade organic matter in an heterogenous and irregular pattern, causing cell collapse, granulation, shrinkage and irregular perforations, but no regular perforations [193] and other chemical and physical degradation features, as also illustrated in taphonomic experiments with added mixture of natural microbial communities [194].

Finally, small dark brown protuberances that consist of 3.4 to 3.65 µm in diameter groups of ovoid vesicles (1 to 1.6 µm, possibly cells) are irregularly spaced and aligned on one external side of a *Navifusa* specimen that is 186 µm long and 27.15 µm wide (figure 3*t–w*). *Navifusa’s* smooth wall is light brown in colour and shows fine folds due to compression. These protuberances are not internal inclusions since they occur on the wall surface, nor a taphonomic feature, nor an ornamentation as they do not occur on other specimens of *Navifusa* nor on other fossils observed here. They are interpreted as possible (bacterial or protist) ectobionts on the cyanobacteria *Navifusa*.

## 4. Discussion

### (a) The 1.78−1.73 Ga McDermott microfossil assemblage

The 1.78−1.73 Ga McDermott assemblage preserves at least 35 organic-walled microfossil mophotaxa, including nine taxa interpreted as eukaryotic (*D. macroreticulata, G. doncooki, L. kulgunica, L.* sp. with a four curved-slit cyst opening, *Pterospersimorphita* sp.*, S. annulare, T. plana*, unnamed form B—a new acanthomorph with dark spines, and the filamentous multicellular *Siphonoseptum* sp.), two probable multicellular eukaryotes (unnamed form C, *J. solubila*), one unambiguous cyanobacteria taxon (*N. majensis*), 10 probable cyanobacteria (*Paleolyngbya, Rugosoopsis tenuis,* six species of *Siphonophycus, Tortunema,* a false branching sheathed filament of a possible oscillatoriale) and several (13) unidentified entities that could be prokaryotic or eukaryotic, (*Cucumiforma* sp., *Leiosphaerida crassa, L. jacutica, L. minutissima, L. tenuissima,* three *L.* spp.*, S. sinica*, *Changchengonema densa*, fragments of long opaque tubes and fragment of fibrous tubes in sheath, unnamed form A). This is a conservative estimate, some of these taxa being considered likely or possibly eukaryotic in the literature.

### (b) The oldest eukaryotes to date

**Figure 6.**
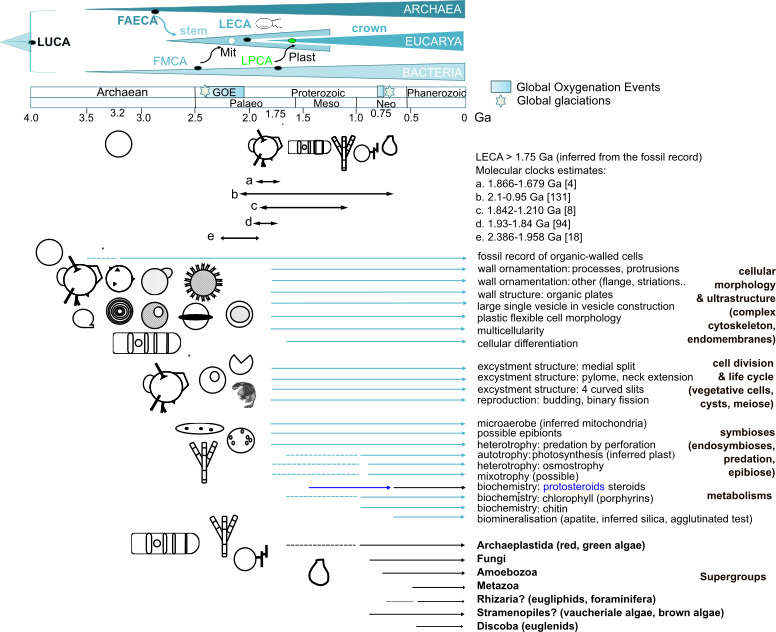
Cellular palaeobiology and implications for eukaryogenesis. Timing of eukaryogenesis inferred from the microfossil assemblage preserved in the 1.78−1.73 Ga McDermott Formation, Tawallah Group, McArthur Basin, Australia. Global oxygenation events (GOE, NOE) and global glaciations are indicated. Summary of the Precambrian record of organic-walled fossil cells, their observed and inferred features of cellular morphology and ultrastructure implying a complex cytoskeleton and endomembrane system, cell division and life cycles, diverse possible symbiotic interactions and metabolisms; and minimum ages of crown eukaryotic supergroups. The minimum age of LECA (Last Eukaryotic Common Ancestor) is >1.75 Ga based on the fossil record reported here and is consistent with estimations from molecular clocks. FAECA: First Asgard Eukaryotic Common Ancestor; FMCA: First Mitochondrial Eukaryotic Common Ancestor. LPCA: Last Plastid Common Ancestor.

This diverse assemblage documents remarkable eukaryotic cellular features (figure 6) such as tubular processes, spiny processes, diverse neck-like or bulbous protrusions and possible flange; the ability to synthesize organic plates to build a cell wall; vesicle in vesicle construction; unusual excystment structure by four curved slits opening; sophisticated cyst opening by a pylome or at the end of a neck-like expansion; simple multicellularity in filamentous and vesicular forms; and predation by cell wall perforation (eukaryovory). These complex morphologies associated with large size indirectly evidence the presence of a complex cytoskeleton and endomembrane system of eukaryotic cells [117,121,178,195]. Complex excystment structures also require a cellular machinery and genetic control of eukaryotic grade. Some of the McDermott microfossils move back the oldest record of eukaryotes, complex excystment structures and simple multicellularity in eukaryotes by at least 100 Ma, and possible eukaryovory by 650 Ma (or 100 Ma if a Ruyang example is confirmed). This assemblage is also remarkable by preserving intracellular thylakoidal compartments in fossil cyanobacteria *N. majensis*, pushing back the fossil record of such endomembranes by more than 1 Ga from the report in the Draken Fm, Spitsbergen [101], and providing direct evidence for the ability to perform oxygenic photosynthesis [95].

Cysts are part of the life cycle of many protists, often associated with sexual reproduction (meiosis, unique to eukaryotes) or protection against adverse environmental conditions [196]. In modern protists, budding and fused vegetative pairs evidence asexual reproduction (as illustrated in some specimens of *Gemmuloides* and *Tappania*) while cyst (as shown by complex excystment structure in *L. kulgunica*, *D. macroreticulata*, *T. plana* and unnamed vesicle *L.* sp.) and colonial stages (possibly in one specimen of *T. plana* with four asymmetrically attached vesicles) may evidence meiosis, often triggered by stress-nutrient starvation, light or salinity change [196]. Cyst opening by medial split occurs here in unornamented vesicles (leiospheres) that could be prokaryotic, such as pleurocapsale cyanobacteria [177,184], or possibly eukaryotic for the larger forms of over 50 or 100 µm in diameter.

The Proterozoic fossil record of symbioses is scarce and probably overlooked but their ecological and evolutionary implications are fundamental and they may lead to crucial transitions in life evolution. In the Neoproterozoic, dark discoidal, semicircular or ovoid structures (0.5−18.4 mm in length and 0.9−3.6 mm in width) were interpreted as possible ectosymbionts (or ectobionts or epibionts) preserved on specimens of the macroscopic (up to 25 mm long) eukaryotic fossils *Tawuia* and *Sinosabellidites* from the 1 Ga Liulaobei Formation, North China [197]. Possible cyanobacterial epibionts on a cyanobacterial filament were reported in silicified stromatolites from the approximately 1.5 Ga Gaoyuzhuang Formation, China [198]. The example illustrated here on *Navifusa* possibly pushes back the record of ectosymbiosis by about 250 Ma (figure 6). Ectosymbiosis is known in prokaryotes such as *Vampirococcus*, which may form piles of up to 10 predatory bacterial cells (500–600 nm diameter) attached to the surface of a bacterial host [199]. Prokaryotes (archaea and bacteria) can also engage in extracellular symbioses with protists [2], such as an ectosymbiotic deltaproteobacteria at the origin of magnetoreception in an excavate protist (Euglenozoa) in marine anoxic sediments [200]. Protist ectosymbionts on protists are also common, and they often involve photosymbionts, for example dinoflagellate ectosymbionts of foraminifera or radiolaria [201] and presumably, small protists may become ectosymbionts on large prokaryotic hosts, although this is probably rarer considering the average size of prokaryotic and eukaryotic cells.

Myzocytosis, or cellular vampirism—the process by which a cell pierces the surface of another cell and sucks out its contents for food [189]—evidences a sophisticated protist feeding behaviour and another type of microbial symbiosis, eukaryovory. The shapes of cell wall perforations may vary from round to oval holes in Neoproterozoic acritarchs [173,192,202] and half-moon holes in 0.75 Ga testate amoebae, Chuar Group, USA [202]. Prior to this study, the oldest known examples were from the 1.15−0.89 Ga Shaler Group, arctic Canada [192]. These perforations were interpreted as predation marks made by protists [192,202–204]. A possible occurrence needing confirmation is reported in the approximately 1.65 Ga Ruyang Group, where an unidentified tube fragment is perforated by some 3.5−4.0 µm ‘lacunae’ [205]. The size and shape of the perforations observed in the McDermott example (0.46–0.84 µm oval holes) are similar to those observed in some taxa from the Chuar Gp and lower Shaler Group. Modern protists such as the vampyrellids amoebae (Rhizaria) pierce the organic walls of algae or other protists or fungi by making one or multiple regular holes on a single prey (e.g. images in [206]), using complex strategies involving enzymatic digestion [207] or sophisticated cytoskeleton implications for the removal of an excised disc from the prey cell wall [189]. This behaviour is found in several different kinds of predators, such as didinid ciliates, colpodellids, colponemids, noctilucoid dinoflagellates and vampyrellid cercozoans and some marine intestinal parasites, and in the recently defined deep-branching supergroup Provora [44,65]. Predatory behaviour also exists in bacteria. Epibiotic predators such as *Bdellovibrio exovorus* stay attached on the bacterial prey cell wall and feed on the cell contents, while endobiotic predators, like *Bdellovibrio bacteriovorus*, grow and reproduce within the periplasmic space of diderm bacteria [208]. The (1.2 µm long) predatory epibiont *B. exovorus* leaves an approximately 160 nm open scar that is not resealed [208]. *Bdellovibrio bacteriovorus* swims with its flagellum and collides with a prey cell, after which it becomes irreversibly anchored via the pole opposite the flagellum by passive protein adhesion and pili active adhesion. It generates a small opening in the prey cell’s outer membrane and peptidoglycan layer, which is ultimately resealed. A mixture of hydrolytic enzymes is applied locally and solubilizes the prey peptidoglycan [209] but the circular entry holes are much smaller (approximately 200−300 nm scar sealing the 100 nm hole [210]) than those of modern protist vampires and those observed in fossils. Based on these observations, the holes perforating a cell wall or sheath observed here are tentatively attributed to predatory eukaryotes, evidencing eukaryovory by wall perforation, possibly by myzocytosis, pushing back the fossil record of this sophisticated heterotrophic predatory behaviour by 650 Ma from the Shaler examples (or by 100 Ma if the Ruyang example is confirmed). Although many modern protist vampires discussed above are not early branching clades, the recently discovered Provora form a new deep-branching supergroup of eukaryotes [64], suggesting that eukaryovory might have appeared early in eukaryotic evolution among small flagellates, as did phagocytosis.

Both possible examples of symbiotic interactions suggest complex and diverse ecological interactions between eukaryotes (eukaryovory) and between a prokaryotic host and eukaryotes or prokaryotes (ectosymbiosis) in the McDermott microbial ecosystem. Interestingly, based on the Proterozoic record known to date, this predatory behaviour seems to target smooth-walled vesicles and tubes and ornamented protists but not process-bearing (spiny) protists, perhaps evidencing their defence mechanism and suggesting a long history of predator–prey interactions. Presumably, symbiotic interactions were abundant in Proterozoic ecosystems and a careful re-examination of the fossil record could reveal more preserved examples.

Except for a few taxa (an unnamed spiny vesicle and an unnamed leiosphere with a complex ‘flower-like’ cyst opening, both interpreted as eukaryotic, and unnamed multicellular taxa that are possible eukaryotes), most of the microfossil taxa reported in the approximately 1.75 Ga McDermott Formation are known in other younger Proterozoic successions worldwide, including in the late Palaeoproterozoic of Australia and China and Mesoproterozoic of Australia, Canada, China, India, Russia and the USA (see recent compilations in [122,123,125]). Their occurrence reported here expands their stratigraphic record by at least 100 Ma, including for the iconic early protist *T. plana* that shows a spectacular plastic morphology and simple multicellularity, and *Dictyosphaera* with a wall made of organic plates and a programmed pylome.

Prior to this work, the oldest unambiguous eukaryotic fossils reported in late Palaeoproterozoic successions occurred in the approximately 1.65 Ga (younger than 1673 Ma dyke in archean basement) Changzhougou Fm and the 1.641.7 ± 1.2 Ma Chuanlinggou Formation (Changcheng Group) from North China (U-Pb zircon age from a tuff layer within black shales) [55,99,211–215], the poorly constrained 1744−1411 Ma Ruyang and Gaoshanhe groups from southern North China [117,179,216–219], the 1653 ± 17 Ma Mallapunyah Formation (McArthur Supergroup) from North-Western Australia [115,118,166,220,221], the 1642 ± 3.9 Ma Limbunya Group, Birrindudu Basin, northern Australia [122,220,221] and the approximately 1630−1600 Ma Semri Group from India [222].

As discussed earlier [117,121,122,178,195], size by itself cannot discriminate between prokaryotes and eukaryotes because there are picoeukaryotes that are 1−2 µm in diameter [223] and rare large square archaea up to 12 µm or 100 µm long filaments with 2−3 µm cells [224] and bacteria up to a few hundred µm or mm in diameter or up to 9 mm in length [162]. However, these prokaryotes do not show the combination of large size and complex cyst opening structures, and wall ornamentation such as organic plates, flange, neck-like expansions and spines or tubular processes that modern protists and some of the fossils reported here do. As discussed above, some prokaryotes can have cells forming membrane-branching protrusions in Lokiarchaeales (e.g. [27,28]) sustained by an actin cytoskeleton but they lack an endomembrane system [30] or elongated cells with a spherical expansion in Hodoarcheales [29] or cell wall protrusions and warts in PVC bacteria [225–227], cellular differentiation in Nostocales cyanobacteria, simple multicellularity in filamentous or colonial forms, internal membranes and compartments in some bacteria [22,228,229] but except for cyanobacteria, these complex prokaryotic cells are very small (less than 1 µm and up to 5 µm for protrusion-bearing Archaea and up to about 10 µm for PVC bacteria), while the larger bacterial and archaeal cells are simple in morphology. Moreover, although morphologically simple and complex cyanobacteria that have peptidoglycan cell walls and polysaccharide sheaths preserve very well as carbonaceous compressions in mudstone (as in this study; e.g. [95,230]), and this could potentially be the case for some other bacteria as well, the fossilization potential of other complex prokaryotes is unknown and might be challenging in a similar preservation mode, especially for most archaea that have a cell coat of glycosylated proteins (the S-layer) coating the lipid membrane instead of a cell wall (although some clades also have a N-glycan layer [224,231]). In another taphonomic window, their fossilization by rapid silicification and preservation in chert might however be possible, as suggested by taphonomic experiments [232].

Once microfossils are identified as eukaryotic, another challenge is to test hypotheses regarding the taxonomic placement of these earliest fossil eukaryotes into stem clades before LECA or stem or crown clades of crown groups after LECA, leading to a debated timing for eukaryogenesis and for the early or late age of LECA.

### (c) Multiple sources of diverging views on the age of LECA

#### (i) A question of definition

There are conceptual differences regarding the defining features of a eukaryote, FECA and LECA [9,15,17,41,233] that impact the interpretation of the fossil record and estimations by molecular clocks. Because there are at least two FECA—one from the Asgard archaea side and one from the alphaproteobacteria side—the archaeal FECA can be called Asgard FECA (‘first descendant—on the eukaryotic side—of the last common ancestor of an Asgardarchaeota lineage and the eukaryote’, [17]) or FAECA (First Asgard Eukaryotic Common Ancestor) and the mitochondrial FECA is called FMCA (‘the first descendant of the last common ancestor of the alphaproteobacteria-related progenitor and the eukaryotes’ or the First Mitochondrial Eukaryotic Common Ancestor) [17]. The cellular features associated with eukaryotes might have evolved at any point on the stem lineages between either the archaeal FAECA and LECA or between FMCA and LECA [9,17]. At the younger end of eukaryogenesis is LECA, the last eukaryotic common ancestor—a modern aerobe flagellate mitochondriate protist with a complex cytoskeleton, nucleus and endomembranes system—but other features such as the gene repertoire of LECA are far from established [17]. These ambiguities also result from recent discoveries on the cellular complexity of some prokaryotes, including 0.5–5.0 µm archaea with appendices and a cytoskeleton [27–29]; one (up to 10  µm) ‘phagocytic-like’ planctomycete bacterium [45]; and of bacterial cellular compartmentalization with organelles, such as thylakoids, magnetosomes and anammoxosomes [229] and other internal membrane structures [228]. These prokaryotes, however, are not capable of phagocytosis or endocytosis [43,200,228]. Genetic material is sometimes located in deep invagination of membranes that are not closed like a nucleus, except perhaps in an anaerobe Atribacteria [234] and in a recently discovered giant (up to 9000 µm for a single cell) *Thiomargarita magnifica* with DNA and ribosomes compartmentalized into a metabolically active, membrane-bound organelle [162]. Such discoveries of huge bacteria [162] or tiny eukaryotes [223] show that exploration without preconceived ideas may reveal new diversity, and this may be true as well for the exploration of the fossil record. It is unclear whether these traits are ancestral or derived in these prokaryotes but still it shows that prokaryotes can have a more complex cellular ultrastructure and morphology and larger size than previously thought, which is somewhat challenging the concept of a bacterial, an archaeal or an eukaryotic cell.

#### (ii) Estimates from molecular clocks

The methods and data used for molecular phylogenies suffer from incomplete sampling and knowledge of modern protist and prokaryotic diversity, although this knowledge is rapidly increasing. This in turn affects molecular clocks that use diverse types of phylogenies, fossil calibrations (with uncertainties on identification and ages) and methods to estimate evolution rates. Estimates for the age of LECA range from 2.39 Ga to 0.95 Ga depending on molecular clocks (1.866−1.679 Ga [4]; 2.1−0.95 Ga [131]; 1.842−1.210 Ga [8]; 2.386-1.958 Ga [18]; 1.93−1.84 Ga [94]; or 1.88–1.5 Ga [78]). Divergent interpretations of the body and molecular fossil record also infer either a possible late Mesoproterozoic LECA [129,140,142] or an earlier LECA ( [119,124,132–134,139]). In addition, some studies propose a possible persistence of stem groups (before LECA) co-occurring with stem of crown groups (after LECA) (e.g. [9,55,119,133,137]). This co-occurrence of stem and crown lineages is known for example in plant or arthropod evolution [235], but whether stem lineages can persist for long or short times is debated (see [235] versus [236]). Moreover, distinguishing stem clades (before LECA) from stem or crown members of crown clades (after LECA) is a challenge ([118,119,122,126,132,133,142] and discussion above). For example, estimates for the primary endosymbiosis of the chloroplast have a minimum age of 1.047 Ga, based on the uncontested body fossil record of stem red alga *Bangiomorpha* [51,127] and have suggested it to be as old as 1.64 Ga based on the multicellular microfossil *Qinshania* [55]. Some molecular clocks also estimate a Palaeoproterozoic age for the primary plastid endosymbiosis (2137–1807 Ma, [18]) or a late-Palaeoproterozoic to early-Mesoproterozoic age (2.28–1.48 Ga [146]; 1712–1387 Ma [237]).

#### (iii) Interpreting the molecular fossil record

The biomarker record has its own challenges as well, one of the strongest being the preservation issue limiting the stratigraphic record where these fossil molecules can be preserved because they are sensitive to temperatures higher than 150°C in fossil oils [238]. Consequently, fossil molecules from oil have a shorter geological record but can document the evolution of biosynthetic pathways of ecologically important clades (e.g. [129,239,240]). Porphyrins (remains of chlorophylls) were found within 1 Ga fossil cells that had been heated up to 180°C by burial [53,230], as well as lipidic thylakoidal endomembranes in 1.75 and 1 Ga cells [95] , suggesting that steroids could perhaps also be detected within protective fossilized cell walls at higher thermal maturity. Structural biopolymers such as polysaccharides and possible chitin can be preserved as well in Proterozoic cell walls and help to elucidate microfossil identities when combined with other morphological and ultrastructural features to minimize the possibility of convergence (e.g. [157,230]). Contamination is another major issue that is now addressed by rigorous lab protocols [238]. Other challenges include sampling bias owing to too few data points for the oldest record [130], taphonomic issues such as in microbial mats where steroids might be less preserved [241] (although this is disputed [239]), or ecological issues such as a low abundance of eukaryotes compared with prokaryotes or a restriction of photosynthetic eukaryotes to some niches [107] (see also discussion in [134,240]). Microbial reworking and oxygen exposure can also blur the biomarker sedimentary record [242]. Finally, the gap of knowledge in modern biochemistry also hampers the interpretation of the sources of fossil biomolecules [240].

LECA had membranes composed of fatty acid chains linked to a glycerol-3-phosphate (G3P) head group via ester bonds [243] and could produce a large diversity of sterols that may have allowed an increased fluidity of its eukaryotic membranes, an important step towards increasing cell size and a possible protection against oxidative stress [37]. Subsequent evolution occurred through differential enzyme losses and specializations in the various eukaryotic lineages in parallel with their divergence from the LECA [37]. LECA likely produced modern-type steroids while some steroid-producing bacteria produce protosteroids and archaea do not produce steroids at all [130]. Early eukaryotes received the capacity to synthesize steroids, an oxygen-dependant process, by LGT from bacteria, probably by the time of the GOE [86,240]. Molecular clocks estimation dates the synthesis of protosterols > 2.31 Ga and of sterols by crown eukaryotes between 1.30 and 2.17 Ga [86].

The interpretation of the biomarker record indicative of eukaryotes is debated. Protosterols are found in rocks from 1.65 Ga [244] and sterols from 800 Ma, with sterols typical of algae from 780 Ma [129,150,240]. Gold *et al*. [86] interpret this late detection of modern sterols as reflecting the ecological expansion of eukaryotic plankton in the Neoproterozoic rather than the origin of crown eukaryotes as interpreted by Brocks *et al.* [129], who suggest that the record of protosterols reflects stem eukaryotes. However, there are alternative ways to interpret the biomarker record and a young LECA does not fit the fossil record or most molecular clocks. Protosterols can also be produced by bacteria and the record of sterol production by crown eukaryotes could be due to sampling bias, with too few data points for the oldest record [130]. Indeed, there are no sterols found before 800 Ma, although there are crown group eukaryotes such as probable fungi [157] and multicellular algae since at least 1 Ga [51–53], implying an earlier age for the primary endosymbiosis of the chloroplast and consequently an even earlier age for LECA. Recently, 1.64 Ga multicellular microfossils with several cellular types were proposed as possible algae [55]. Previous palaeontological studies also suggested an early origin for the Archaeplastida [51,245] but these interpretations are contested. Some molecular clocks estimate a Palaeoproterozoic age for the primary plastid endosymbiosis (2137–1807 Ma [18]) or a late-Palaeoproterozoic to early-Mesoproterozoic age (2.28–1.48 Ga [146]; 1712–1387 Ma [237]), and an early origin of fungi as well (1759–1078, Ma [18]). Finally, as cited above, based on a size- and trait-based ecosystem model, the preserved microfossil record suggests an active eukaryotic ecosystem conducting osmotrophy, photosynthesis and phagotrophy [156].

A compromise could be that the stem of crown eukaryotes (after LECA) would still produce protosterols (as suggested in [55]), or a diversity of lipids that later specialized in diversifying lineages after LECA (as suggested in [37]). Indeed, sterols are essential in membranes of modern protists where they form lipid rafts with sphingolipids [23]. An optimal composition of plasma membrane allows reduction in the energetic costs of membrane compartmentalization and reconfiguration. Membrane thermodynamics depends on a complex interplay of proteins and lipids and is highly sensitive to the heterogeneous distribution of lipids that promote curvatures, fluidity, vesicle formation and trafficking, activity and permeability of ion-channels and excitability of protist cells [23]. Moreover, modern-type steroid biosynthesis and associated metabolites are also important in establishing a variety of aerobic metabolisms, crucial for balancing the ecological role of bacteria and eukaryotes in response to late Proterozoic environmental changes [130].

#### (iv) Interpreting the body fossil record

Challenges in identifying eukaryotes among microfossils can be owing to biases of the fossil record (most lifeforms are not fossilized) and of the geological record (most rocks that could preserve fossils have been altered or destroyed by geological processes), sampling bias [125,151], preservation, possible convergence with prokaryotes, unseen diversity of picoeukaryotes as illustrated in modern environments [223], lack of observable eukaryotic traits (for example in smooth-walled spheres) and gaps in biological knowledge required to recognize taxonomically relevant traits in fossils. As discussed above but often miscited in the literature, size is not a distinctive criterion by itself (e.g. [117,195]). Morphologically simple (unornamented) 50–300 µm diameter organic-walled vesicles (or acritarchs) that can be abundant in Archean mudstones [136] or in most Proterozoic samples (thousands of specimens, including this study) may or may not be eukaryotic [115,119,135–137,246] but large and morphologically complex organic-walled vesicles with wall ornamentations or complex expansions, or plates or complex programmed cyst opening structures are most certainly eukaryotic fossils [117–119,121–123,125,132,133,178,180] because these features require a cytological machinery of eukaryotic grade and are unknown in prokaryotes. The same is true for multicellular filamentous forms, which are usually interpreted as prokaryotic when simple (although they could include some eukaryotes), and that are interpreted as eukaryotic when they show a level of complexity with cell size, cellular differentiation or ornamentation or division mode unknown in prokaryotes (e.g. *Bangiomorpha* [51]; *Siphonoseptum* [122]; and *Qingshania* [55]). Once microfossils are identified as eukaryotic, another challenge is to place them on the eukaryotic tree of crown eukaryotes after LECA or as stem eukaryotes before LECA. Regardless of their identity, these organic-walled fossils, once well dated and convincingly identified as eukaryotic, provide important information about the evolution of biological innovations or characters in early eukaryotes [89,115,118,119,132,133,137] and their taxonomic diversity and their morphological disparity [122,123,125,247].

Besides debates on taxonomic identification that can be blurred by taphonomic processes such as phosphatization, uncertainties on the age of microfossils may also occur in areas with complex geological history where discontinuous lateral exposure prevents robust stratigraphic correlations. These challenges are illustrated by the case of putative crown-group red algae reported in the Vindyan Supergroup of central India [54]. *Rafatazmia* are septate filaments with putative pit plugs that are not clearly evidenced, casting doubt on a red alga identity [126], and *Ramathallus* forms multicellular thalli resembling younger Ediacaran fossil red alga *Thallophyca* [139]. The age of these fossils is proposed to be 1.6 Ga [54] but could be as young as 1.07 Ga, depending on correlations [127].

The fossilization potential of most prokaryotic cells is unknown, except for cyanobacteria and possible iron-oxidizing and sulfur-oxidizing bacteria (reviews in [89,90,115,174,248]), but the extant prokaryotic complexity calls for refining taxonomic criteria to discriminate eukaryotic from prokaryotic microfossils in the fossil record by combining morphological traits with ultrastructural and chemical information and the environmental context [53,95,101,157,180,230,249,250]. Similarly, many protists are small and/or have simple morphologies (undecorated cell walls) or do not produce cysts or recalcitrant vegetative structures that could be preserved and identified in the fossil record. This could be the case for LECA and other small flagellates that are the most abundant protists and that may not have left a fossil record.

Micro- to nano-scale analyses of the morphology, ultrastructure and chemistry of these fossil cells combined with microbiology and experimental taphonomy of modern microorganisms (e.g. [126,194]) permit better understanding of the fossilization processes. This information allows us to identify the fine details of eukaryotic cellular features, metabolisms, ecological interactions, and in some cases to confirm the identity of specific microbial clades. In particular, the ultrastructure of cell walls and intracellular organelles, combined with chemistry and morphology, can provide critical clues to the fossil identity and in some cases, their placement in stem or crown groups [53,95,157,180,230,249–253]. Fossil cells sometimes include intracellular inclusions (ICI) that have been variably interpreted as nuclei, diverse compounds storage, collapsed cytoplasm or chloroplasts (e.g. [126,254]). These ICI can preserve fossil molecules indicative of a metabolism, such as porphyrins remnant of chlorophyll and indicative of photosynthesis in approximately 1 Ga multicellular branching algae *Arctacellularia* [53] and approximately 1 Ga Stigonematacean cyanobacteria *Polysphaeroides* [230]. The latter study shows that the presence of an ICI in a fossil cell is not by itself indicative of eukaryotic affinity, nor a chloroplast or a nucleus. One possible mode of ICI formation is nicely illustrated in the case of desiccated specimens of the cyanobacteria *Stigonema* from an herbarium collection, where cells preserve shrunken cytoplasm still containing thylakoids [230]. Taphonomic experiments in water, although not mimicking natural fossilization processes in sediment, suggest the possible preservation of nuclei, chloroplasts and condensed cytoplasm of algae [126]. Other experiments on cyanobacteria and eukaryotic algae also in water but with a natural mixture of microbial decomposers show differential preservation of polysaccharide sheath and cell walls [194]. In figure 1 of that paper [194] , the degraded cytoplasmic content of the discoidal cells of the red alga *Bangia* is still visible. Unfortunately no ultrastructural analyses with electron microscopy was performed in either study to evidence the preservation or degradation of subcellular features, such as in naturally fossilized microfossils [95,101,180,230].

The protists *Shuiyousphaeridium* (ornamented with furcated processes and polygonal ridges [180]) and *Dictyosphaera* from the approximately 1.65 Ga Ruyang Group, China have organic walls made of imbricated bevelled plates [179,180], suggesting their synthesis and placement in the wall thanks to an endomembrane system [180]. Porter & Riedman [142] suggested instead that the plates of *Shuiyousphaeridium* resulted from the wall's physical properties, which displayed cracking similar to desiccation cracks (and so were not actually plates), as opposed to the case of *Satka favosa*, another Palaeoproterozoic to Mesoproterozoic protist that also has a wall made of plates and showing cyst opening by medial split (e.g. [178,180]). However, this alternative interpretation [142] seems difficult to reconcile with the wall structure and multilayered ultrastructure visible with electron microscopy [180,195]. These complex protists may contain ICI that have been tentatively interpreted as possible nuclei [126]—or condensed cytoplasm in the process of forming cysts [254]—of total-group eukaryotes (stem and crown groups) [122,142] or of possible algae [179]. *Tappania plana*, which has an amazingly flexible morphology implying both a metabolically active organism that can be multicellular and the presence of a dynamic cytoskeleton, as illustrated in the McDermott Formation that preserves the oldest protists reported to date (this study)—may also include an irregular ICI interpreted as condensed cytoplasm [117,133,178,180]. Many other Proterozoic ornamented and simple microfossils contain ICI. Proterozoic cyanobacterial cells as old as 1.75 Ga ([95]; this study) and younger (e.g. [101]) may preserve endomembranes in various taphonomic windows. These observations suggest that the Proterozoic fossil record should be probed with micro- and nano-scale analyses of the morphology, ultrastructure and chemistry of microfossils, as underlined above, to search for the possible intracellular preservation of chloroplasts or other organelles and molecules, and for structural features and composition of the cell walls of early fossil cells that could provide crucial information to discriminate prokaryotes from eukaryotes (e.g. [101,180,230,249,255]) and to decipher the early record of eukaryotes and their identification as stem or crown clades (e.g. [53,192]). The identified and well-dated body and molecular fossil record can then be used for calibrating molecular clocks constructed on phylogenies. These age estimates and order of branching events can then inform back on possible clades that could be searched for in the geological record.

### (d) Stem and crown eukaryotes among the McDermott microfossils?

Accepting that there are unambiguous eukaryotes within the McDermott assemblage, the question of their stem or crown identity then still needs to be addressed. These protists could be interpreted as (i) stem anaerobe eukaryotes (before LECA) without mitochondrion, with or without a nucleus, but with a cytoskeleton and endomembrane system; (ii) stem microaerobe eukaryotes (before LECA) with a mitochondrion, a cytoskeleton and endomembrane system and presumably a nucleus; (iii) stem members of crown eukaryotes after LECA; (iv) crown members of crown eukaryotes after LECA; (v) a combination of stem groups (before LECA) persisting along crown groups (after LECA); (vi) undifferentiated total group eukaryotes. As discussed above, future detailed analyses could reveal informative features to better constrain the identity and palaeobiology of these earliest protists known to date, but the morphology and palaeoecology reported here might already permit the testing of some hypotheses.

Butterfield [132] proposed that ‘large (>5−10 µm) morphologically complex microfossils can be used as a clear and conservative measure of crown-group eukaryoticity’ because 1) their stratigraphic and global distributions are difficult to reconcile with stem-group status, 2) many microfossils have a morphology present in an already diversified range of crown eukaryotes, and 3) fossils with similar morphology such as process-bearing acritarchs present at younger Phanerozoic ages are considered crown-group because they co-occur with identified crown groups. Despite the recent discoveries of prokaryotic cellular complexity discussed above that urge caution when interpreting the fossil record based only on morphology—including archaea and bacteria with protrusions or compartments or large cell size—there is still a gap with the level of eukaryotic complexity shown by most large ornamented microfossils with programmed cyst opening. With these criteria, all the simple undecorated small protist cells that are the most abundant eukaryotes in modern ecosystems, such as small flagellates and LECA, would unfortunately be unrecognized based on morphology alone if they were preserved in the Proterozoic fossil record. Identification of crown-group eukaryotes that are accepted by most is extremely rare in the Proterozoic record but even those fossils that are used as calibration points in molecular clocks suggest a Palaeoproterozoic LECA, older than the Late Mesoproterozoic or early Neoproterozoic (see above). Based on their reading of the fossil record, Cohen & Kodner [134] suggested that eukaryotes were likely aerobic and established in Proterozoic ecosystems, at least by the Mesoproterozoic. In another interpretation of the body and molecular record, Porter *et al*. [98] considered the possibility that fossil eukaryotes were stem clades living in anoxic environments until the appearance of LECA in the late Mesoproterozoic/early Neoproterozoic, close to the age of fossil crown Archaeplastida. Recently, Riedman *et al*. [122] proposed that stem and crown eukaryotes cannot be distinguished in the early fossil record, so they consider all eukaryotic fossils as members of the total group eukaryotes.

However, the morphological complexity and disparity and cyst opening structures, *combined with* large cell size, and possible predatory behaviour of some of the McDermott protists clearly differ from that inferred for early stem-eukaryotes that would resemble their archaeal ancestor or even for LECA that resembled a small flagellate excavate. In addition, based on the presence of filamentous *Siphonophycus* benthic mats and thylakoid-bearing cyanobacteria, some molecular oxygen must have been available in the supratidal to intertidal very shallow marine niches where the McDermott protists lived. As discussed above, nanomolar concentration of molecular oxygen is sufficient for early and many modern mitochondriate eukaryotes to thrive and to synthesize sterols. Taken together, these observations permit the hypothesis that some of the McDermott protists had acquired a mitochondrion in addition to a dynamic cytoskeleton, an endomembrane system and a nucleus, and evolved after LECA, perhaps living along persisting stem lineages. To date, these protists cannot yet be placed in modern crown groups either because they are stem members of these crown groups and lack some distinctive morphological characters, or because they do not preserve diagnostic features. Future analyses of their chemistry and ultrastructure might help to identify crown groups, for example by evidencing the presence of chloroplasts or other organelles or intracellular features and molecular remnants unknown in prokaryotes.

The alternative hypothesis that large decorated cells with programmed opening cyst structures or walls made of organic plates or appendices, or large multicellular and ornamented filaments or vesicles, or microbial cells capable of complex behaviour such as eukaryovory (possibly protist vampirism), which thrived in or close to microoxic niches, would lack mitochondria (and thus were anaerobe), and/or cytoskeleton, and/or endomembranes and/or nucleus and would have evolved before LECA, which was a small excavate-like protist, followed by a diversification of crown clades that would reinvent these complex features seems less parsimonious and unlikely. Even though convergence is common in evolution and these characters can be polyphyletic, it is plausible that some of these features might have appeared only once LECA and crown groups had evolved all the required gene pool and cellular ultrastructural machinery. A possible example is the case of simple multicellularity that exists in the three domains of life (e.g. [162,224,256]) and presumably could appear before LECA in stem eukaryotes, but not complex multicellularity that is also polyphyletic but unique to some crown eukaryotes [257]. This is a hypothesis that could be tested by investigating the molecular phylogenies of specific traits observed in fossils cells, such as the synthesis of plates and construction of cell walls, of particular cell wall ornamentation and expansions, of cyst stages and various cyst opening structures, the combination of large cell size and these features, of complex behaviours like eukaryovory and myzocytosis, and more, to evaluate their possible stem or crown origins.

### (e) Earliest eukaryotes in a possible micro-/nano-oxic niche

The McDermott Formation was deposited in intertidal to supratidal arid evaporitic environments with fluvial input in an intracontinental basin. Marginal environments such as estuarine or intertidal mudflats have been noted previously for their favourable conditions for preserving organic-walled microfossils including eukaryotes in the late Mesoproterozoic 1.03 Ga Mbuyi-Mayi Supergoup, Democratic Republic of Congo, deposited in an intracontinental basin [191] and 1.1 Ga El Mreiti Group, Mauritania [97,183], the late Mesoproterozoic–early Neoproterozoic 1.1−0.9 Ga Shaler Group, Canada [155] and the late Palaeoproterozoic >1642 ± 3.9 Ma Limbunya Group from the Birrindudu Basin, northern Australia [122]. It could be that nutrient input from fluvial source or transport/habitat from continental or estuarine aqueous environments enriched near-shore marine assemblages where these assemblages include rare unique eukaryotic taxa unreported elsewhere, although the other ubiquitous taxa imply connections between marine basins. Some of these successions also occurred in intracontinental basins that avoided euxinic conditions prevailing in open-marine basins [163]. However, a recent study suggests that the fossil record suffers a strong sampling bias [125] so it is premature to distinguish any pattern.

Based on trace elements and mineralogical analyses [163,168], the approximately 1.75 Ga McDermott Formation studied here was deposited in a mostly anoxic–suboxic near-shore marine environment and Fe speciation data suggest more heterogenous oxic/anoxic ferruginous conditions but no euxinia [169]. As described above, this formation preserves unambiguous cyanobacteria preserving thylakoids that directly indicate the local and possibly fluctuating production of molecular oxygen by cyanobacteria. The overlying 1.73 Ga Wollogorang Fm was an euxinic open-marine basin with anoxia in the photic zone [163,168], consistent with the presence of biomarkers of strictly anaerobic photosynthetic green sulfur bacteria [172] and no eukaryotic steroids, so it is possible that euxinic conditions prevented eukaryotic primary producers from spreading in these habitats owing to the unavailability of nutrients sequestered in pyrite (e.g. [107,163]). These observations support the view that the heterogenous low oxygen conditions of the Proterozoic did not prevent eukaryogenesis and that the early protists reported here thrived in microoxic near-shore niches close to cyanobacterial mats. The delay in the onset of euxinia in specific intracontinental basins compared with open-ocean basins in the Palaeoproterozoic, would have provided an important ecological niche for N_2_-fixing microbes and for the early evolution of eukaryotes [168].

Fossil assemblages preserve microfossils living at different water depths of the water column and on and into the sediment, so their co-occurrence might not necessarily indicate similar physiological requirements such as a microoxic niche. Moreover, it is difficult to assess the benthic or planktonic habit of *Navifusa*, although it resembles the modern benthic oval-shaped *Cyanothece* [177]. The same is true for the McDermott protists described here. However, the complexity of morphologies, cyst opening structures and possible complex behaviour, combined with large cell size, support the hypothesis of crown microaerobe eukaryotes probably living along persisting stem lineages, close to thylakoid-bearing *Navifusa* and photosynthetic mats in a very shallow (supratidal to intratidal) coastal photic niche. If this hypothesis is correct, the data presented here suggest a minimum age of approximately 1.75 Ga for the endosymbiosis of an alpha-proteobacterium ancestor of the mitochondrion, and also for the evolution of microaerobe mitochondriate crown eukaryotes with a complex cytoskeleton and endomembrane system, cyst opening structure and vegetative cells indicative of a life cycle, simple multicellularity, heterotrophy by possible eukaryovory and possible osmotrophy, diverse complex ecological and symbiotic interactions between eukaryotes and eukaryote and prokaryotes, and thus for LECA (figure 6). This does not prevent the co-occurrence and persistence of some stem lineages (before LECA) in the assemblage, together with protists that are stem lineages of crown groups or members of crown groups diversifying after LECA. Such association of stem and crown taxa is well known in the younger geological record of plants and invertebrates [235]. Following the proto-mitochondrial endosymbiosis or acquisition (whether it happened at an early, mid- or late stage of eukaryogenesis), stem mitochondriate eukaryotes predominantly inhabited (micro) oxic environments [16]. It may have taken many more adaptations in stem lineages to have fully integrated mitochondria as in LECA [38], although recent work on plastid endosymbiosis suggests that such integration can happen more rapidly than previously thought [2].

An early LECA is consistent with the fossil record of late Mesoproterozoic multicellular algae (1.05 Ga *Bangiomorpha pubescens* [51]; 0.95 Ga *Proterocladus antiquus* [52]; 1.03–0.95 Ga *Arctacellularia* [53]), probable early Neoproterozoic fungi (1.01−0.89 Ga *Ourasphaira giraldae* [157]), possible late Palaeoproterozoic multicellular algae (1.63 Ga *Qingshania magnifica* [55]) and several molecular clocks suggesting the Palaeoproterozoic origin of Archaeplastida [18,146] and an earlier LECA, as discussed above. Strassert *et al*. [18] suggested that oligotrophic Proterozoic waters could have selected for the increased fitness that mixotrophy provided and that is still common in green unicellular algae, so phagotrophy could have persisted alongside phototrophy for long evolutionary times in Archaeplastida. Early predatory protists could also feed on bacteria and on other eukaryotes like modern Provora by nibbling or by perforating and sucking out the cytoplasm of their prey, as inferred by perforated fossil walls (this study). Predation has been suggested as one possible cause for biological novelty such as multicellularity and cell coverings [202–204], intracellular armor, weaponry, parasitism and photosynthesis by endosymbiosis [44], and possibly triggered by the rise of algae in the Tonian [258]. The McDermott assemblage suggests that this selective pressure may have started earlier than previously assumed in eukaryotic evolution. This is consistent also with ecological modelling suggesting a large biomass of eukaryotes active with phototrophy, osmotrophy, phagotrophy and mixotrophy in Proterozoic marine ecosystems [156].

## 5. Conclusions

The McDermott microfossils contribute to improving our understanding of life in late Palaeoproterozoic oceans, and in particular, of the early evolution of eukaryotes and cyanobacteria. Collectively, this late Palaeoproterozoic assemblage evidences a microbial ecosystem with cyanobacterial mats and thylakoid-bearing cells, diverse cells with variable size and wall textures, various stages of life cycle, multicellular eukaryotes, ornamented protists showing some morphological disparity, protists with tubular and other expansions that might have been osmotrophic, and the possible presence of predatory protists feeding on protists by wall perforation as well as possible epibionts on cyanobacteria. These fossil cells evidence an earlier diversification of eukaryotic morphological disparity and feeding strategies and behaviour, suggesting a more complex ecosystem and a more significant role for early protists than previously assumed. This eukaryovory behaviour mostly targeted smooth-walled vesicles and ornamented protists but not process-bearing (spiny) protists, suggesting a long history of predator–prey interactions and adaptations. Most certainly, these early protists had already developed several of the cellular innovations required for the emergence of eukaryotic excitability [23], such as endomembranes and mitochondria serving as ionic compartments and intracellular capacitors, a flexible and adjustable cell membrane, a larger cell size and new strategies for sensing. The cellular complexity as well as possible sophisticated predatory behaviour of the protists documented by the McDermott assemblage allow us to hypothesize that some of them were aerobe and mitochondriate crown eukaryotes (possibly stem and crown members of crown groups) probably co-existing with stem groups in very shallow coastal microoxic environments. The new data presented here also suggest an early LECA, with a minimum age of approximately 1.75 Ga, and imply an earlier eukaryogenesis (figure 6).

Communities of microbial cells interact continuously and closely at the scale of a few hundreds of µm cellular aggregates and at cellular scale. Microbial symbioses occur between prokaryotes (e.g. [199]), between protists [259], and between prokaryotes and protists [2]. Endosymbiosis for the acquisition of the mitochondrial ancestor had already happened by the time of the McDermott microbes, which also had other symbiotic interactions such as possible eukaryovory and ectosymbiosis. These microbial interactions, depending on environmental conditions, may become facultative or obligatory, and may be more often beneficial than detrimental [260] although this is difficult to estimate [261]. They have an important role during microbial evolution in regulating populations, providing food and energy, allowing adaptation to previously inhabitable niches and sometimes leading to important transitions or turning points in evolution such as eukaryogenesis, as already proposed in Lynn Margulis’ seminal paper [262], perhaps in a redox-stratified photosynthetic mat [39], and such as the primary plastid endosymbiosis in freshwater environment [47]. An early LECA implies an older eukaryogenesis in the early Palaeoproterozoic or perhaps the Archean [119,135–137,246], once the ancestors of the Asgard and the alphaproteobacterial lineages and possibly other bacteria involved in this ancient symbiotic affair appeared and some molecular oxygen was available in microbial mats.

Whatever the debates about the age of LECA and interpretation of the fossil record as stem/crown or total group eukaryotes, every discovery of new Proterozoic fossil assemblages seems to reveal a significant level of diversity and disparity for early eukaryotes that shows no clear pattern through most of the Proterozoic before the Cryogenian, pushing back the minimum age of eukaryotes and suggesting a long evolutionary history that precedes the fossil record known to date ([55,122,123,125]; this study).

To better understand the origin and evolution of early eukaryotes and the pattern, timing and causes of their diversification and their symbiotic interactions, future work should focus on (i) the discovery of new microfossil assemblages from more Proterozoic and possibly Archean successions in well-dated and constrained palaeoenvironments; (ii) the careful re-examination of the fossil record to reveal more diversity (to retrieve rare or overlooked forms) or less diversity (if large populations reveal intraspecific variability formerly interpreted as distinct taxa) and to evidence past microbial interactions—this may also require increasing the number of analysed samples per succession; (iii) micro- to nano-scale analyses of the morphology, ultrastructure and chemistry of fossil cells to improve their interpretation as eukaryote and possibly as stem or crown clades—this requires evidencing preserved cell wall construction, intracellular organelles such as plastid and endomembranes, molecular remains indicative of metabolisms, taxonomically informative molecules such as pigments, proteins, polysaccharides and sterols, possibly associated with isotopic signatures (multivariate statistical analyses of these data and machine learning or other approaches may also help to test hypotheses on identification); (iv) realistic taphonomic experiments using modern microbial organisms from early-diverging clades to test their preservation potential in different contexts (although it is impossible to perfectly mimic fossilization processes); (v) more fundamental research on the morphology, ultrastructure and chemical and genetic composition and ecological interactions of modern protists, archaea and bacteria to better understand modern microbial diversity and evolution; (vi) the phylogeny and evolution of the genetic, enzymatic and ultrastructural machinery required for the evolution of possible eukaryotic cellular features preserved in fossil cells, such as diverse cyst opening structures, organic plate synthesis and arrangement, cell wall construction and deformation, diverse types of cell wall ornamentations and expansions, endomembrane synthesis and arrangement, synthesis of biopolymers, complex behaviour such as myzocytosis, a combination of both large size and some of these features, diverse cellular division modes, and more.

This work illustrates how the study of early fossil cells (cellular palaeobiology) can complement the work of microbiologists and provide unique insights into the diversification of ecological interactions in deep time and into some of the fundamental turning points in biospheric evolution: the evolution of oxygenic photosynthesis, the birth of eukaryotic cells and plastid endosymbiosis.

It also documents one possible evolutionary path of a stable biosphere on a habitable planet. Although evolutionary paths are not predictable, life starts microbial, diversifies and remains dominantly microbial. Microbial ecological interactions are ancient, ubiquitous and inevitable, and they inevitably lead to evolutionary novelties that may affect the whole biosphere and planet and may even become detectable remotely.

## Data Availability

Fossil material is hosted in the collection of the Early Life & Traces Evolution-Astrobiology laboratory at the University of Liège, Belgium, and is accessible upon reasonable request.
